# Simultaneous Coproduction of Xylonic Acid and Xylitol: Leveraging *In Situ* Hydrogen Generation and Utilization from Xylose

**DOI:** 10.1002/cssc.202401651

**Published:** 2024-12-27

**Authors:** Ali Awad, Anil H. Valekar, Kyung‐Ryul Oh, Fajar Prihatno, Jaehoon Jung, Ajaysing S. Nimbalkar, Pravin P. Upare, Ji Hoon Kim, Young Kyu Hwang

**Affiliations:** ^1^ Green Carbon Research Center Korea Research Institute of Chemical Technology Daejeon 34114 Republic of Korea; ^2^ Department of Advanced Materials and Chemical Engineering University of Science and Technology Daejeon 34113 Republic of Korea; ^3^ Department of Chemistry University of Ulsan Ulsan 44776 Republic of Korea; ^4^ Activon Ltd. Ochang-eup, Cheongwon-gu, Cheongju, Chungcheongbuk-do 28104 Republic of Korea; ^5^ Chemical Process Solution Research Center Korea Research Institute of Chemical Technology Daejeon 34114 Republic of Korea

**Keywords:** Xylose, Xylonic acid, Xylitol, Dehydrogenation, Hydrogenation

## Abstract

Pentose oxidation and reduction, processes yielding value‐added sugar‐derived acids and alcohols, typically involve separate procedures necessitating distinct reaction conditions. In this study, a novel one‐pot reaction for the concurrent production of xylonic acid and xylitol from xylose is proposed. This reaction was executed at ambient temperature in the presence of a base, eliminating the need for external gases, by leveraging Pt‐supported catalysts. Initial experiments using commercially available metal‐supported carbon catalysts validated the superior activity of Pt. However, a notable decline in recycling performance was observed in Pt/C, which is attributed to the sintering of Pt nanoparticles. In contrast, the synthesized Pt‐supported ZrO_2_ catalysts exhibited enhanced recycling performance because of the strong metal–support interaction between Pt and the ZrO_2_ support. Furthermore, mechanistic insights and density functional theory calculations show that product desorption involves a significantly higher energy barrier compared to substrate adsorption and hydrogenation, highlighting an efficient transfer hydrogenation mechanism leading to equivalent yields of both xylonic acid and xylitol. This study introduces a promising approach for the simultaneous production of sugar‐derived acids and alcohols, with implications for sustainable catalysis and process optimization.

## Graphical abstract

One‐pot synthesis of xylitol and xylonic acid has been proposed in this research work through in situ oxidation and reduction of xylose under ambient reaction conditions. This reaction takes place at room temperature in the presence of a base, eliminating the need for external gases by utilizing Pt‐supported catalysts, offering potential benefits for sustainable catalysis and process improvement.

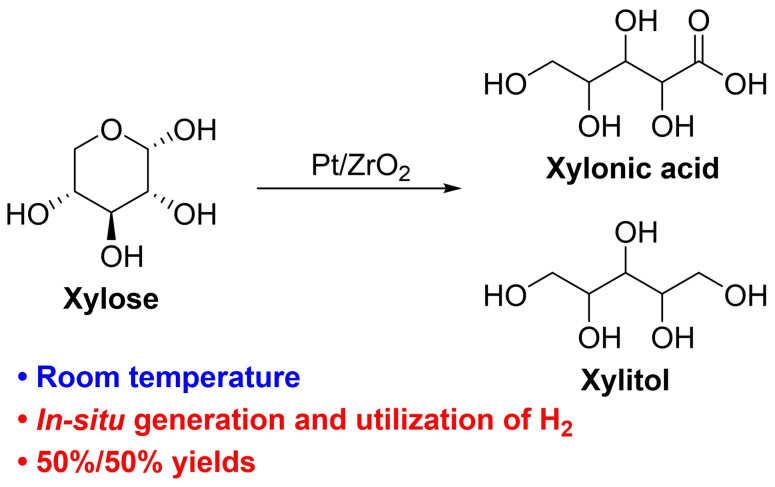

## Introduction

Lignocellulosic biomass, a potential feedstock for the chemical industry, has been the focus of substantial research and developmental efforts in recent years because of its promising properties but challenging processing.^[1]^ It is primarily composed of cellulose, hemicellulose, and lignin. In particular, depolymerization of hemicellulose facilitates a series of reactions, which produces xylose, a precursor to numerous valuable products.^[6]^ This conversion process exemplifies the potential of second‐generation bio refineries, which primarily use lignocellulose and generate sugar alcohols from xylose for use in the food, pharmaceutical, and cosmetics industries.^[7]^ In addition, the oxidation of xylose produces organic acids, which have widespread applications across various industries such as food, pharmaceuticals, detergents, polymers, textiles, and cement retarder additives.^[9]^

Among the sugar alcohols, xylitol is recognized as one of the top 12 bio‐based compounds by the United States Department of Energy (DOE) because of its high sweetening power, low caloric content, and versatile applications in both food and pharmaceutical products.^[11]^ The global xylitol market, which reached nearly 900 million euros in 2021, is projected to grow at a rate of 5.1 %, reaching an estimated value of 1,200 million euros by 2027.^[12]^ Xylitol is in high demand because of its effectiveness as a precursor in the production of valuable products such as ethylene glycol,^[13]^ propylene glycol,^[14]^ lactate,^[15]^ polyesters, and xylaric acid.^[16]^ The conventional production method of xylitol involves the catalytic hydrogenation of xylose using Raney nickel,^[18]^ which, despite its industrial significance, presents challenges such as pyrophoricity, formation of poisonous by‐products, deactivation, and leaching.^[19]^ Therefore, noble metals including Pt, Pd, and Ru have been explored for the catalytic hydrogenation of xylose to xylitol,^[20]^ with Ru emerging as the most efficient catalyst in terms of activity and stability in water.^[21]^ In addition, Co,^[24]^ Ni,^[25]^ Fe,^[26]^ and Sn^[27]^ have been employed as active promoters to enhance the stability of active metals in the hydrogenation reactions. However, because of the necessity for high hydrogen pressures and elevated reaction temperatures, there is a compelling need for a more sustainable alternative method for the production of xylitol from biomass, such as operations at room temperature and ambient pressure.

D‐xylonic acid, which is identified by the DOE as one of the top 30 high‐value chemicals, is a crucial component in the preparation of 1,2,4‐butanetriol, which can be used as a precursor for the production of plastic monomers or medicines.^[28]^ Currently, it is used to enhance concrete dispersions,^[30]^ serves as an efficient biocatalyst for organic transformations,^[31]^ and has applications in the food, pharmaceutical, and agricultural industries. Despite significant research, microbial synthesis of xylonic acid via *Pseudomonas* as reported by Lockwood and Nelson remains the most common method.^[32]^ Subsequent studies have demonstrated that the oxidative metabolism of xylose over archaea and bacteria can yield xylonic acid. Because of the nature of the biocatalytic process, it typically exhibits low productivity (0.3–5 g L^−1^ h^−1^) under mild reaction conditions (<40 °C, pH 5–7), necessitating prolonged reaction time and large equipment volumes. In addition, multistep separation processes, including acidification, esterification, distillation, and hydrolysis, are essential.^[33]^ Recently, the chemical synthesis of xylonic acid has attracted attention as a potentially promising alternative because of its higher reaction rates.^[34]^ Gold‐based catalysts have been used for the oxidation of approximately 0.6 wt % of xylose to xylonic acid at 100 °C–150 °C and oxygen pressures of 20–50 bar.^[35]^ These studies have shown that particle size, electronic properties of Au, acidic and basic properties of the support, and the overall reaction pH are important parameters to enhance the overall productivity of the reaction.^[36]^ However, commercial production of xylonic acid has not yet been developed due to the production of numerous oxidizing enzymes by bacterial strains during the reaction and the low xylonic acid accumulation rates and yields from engineered yeast strains.[^10^, ^38^] Furthermore, harsh reaction conditions, low productivity, and rapid catalyst deactivation under highly alkaline conditions impede the practical applications of chemical oxidation of xylose to xylonic acid.

Moreover, using liquid hydrogen sources instead of gaseous H_2_ in transfer hydrogenation represents an efficient and environmentally friendly strategy for synthesizing fuels or chemicals from feedstocks such as CO_2_ and biomass.^[39]^ Various catalytic systems have been developed employing hydrogen sources such as isopropanol, formic acid, and glycerol. Our research group has investigated simultaneous conversion as an advanced approach, wherein two reactants, including the hydrogen source, are transformed into valuable products such as formic acid and lactic acid.^[42]^ Another significant benefit of this system is that increasing the concentration of reactants can enhance the productivity of both products because the elevated dehydrogenation rate facilitates the transfer hydrogenation process.^[43]^ Therefore, simultaneous conversion, which integrates transfer hydrogenation, offers benefits both environmentally and economically.

**Scheme 1:**
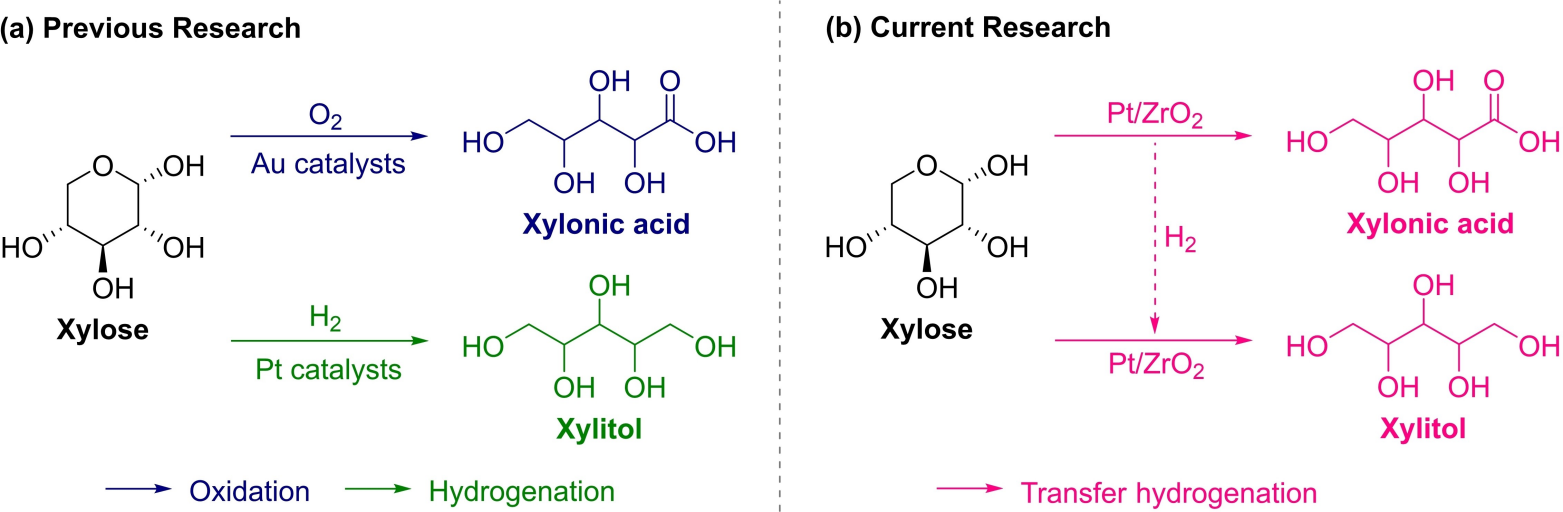
(a) Two existing distinct processes for the oxidation of xylose to xylonic acid and the hydrogenation of xylose to xylitol. (b) Proposed process for the one‐pot coproduction of xylonic acid and xylitol from xylose at room temperature.

Previous studies have shown that the synthesis of xylonic acid and xylitol via the oxidation and hydrogenation of xylose, respectively, requires elevated temperatures (>100 °C) and high external hydrogen and oxygen pressures (>10 bar).^[35]^ These harsh conditions pose a significant barrier to the sustainability of these crucial reactions. Moreover, the use of low xylose concentrations (<0.5 M) in both processes limits the overall volumetric productivity of the system. Therefore, the simultaneous synthesis of xylonic acid and xylitol under mild reaction conditions offers a more sustainable and cost‐effective alternative. Here, we propose a novel approach to enhance the efficiency of transfer hydrogenation through a one‐pot reaction, which involves the *in situ* generation of hydrogen via xylose dehydrogenation and its subsequent use for the hydrogenation of xylose to xylitol. This process employing a Pt catalyst at room temperature is illustrated in Scheme 1. The use of mild reaction conditions, which allows the full conversion of highly concentrated xylose (30 wt %, 2 M in water) into equivalent quantities of xylonic acid and xylitol, is a significant feature of this method. Furthermore, effective separation between carboxylic acid salt and a neutral compound can be achieved using bipolar membrane electrodialysis, as published recently by our research group.^[44]^ Density functional theory (DFT) simulation results indicate that the desorption of both xylonic acid and xylitol represents the most energetically demanding step, thereby elucidating the exceptional hydrogen transfer efficiency (100 %) within this system. Furthermore, the demonstrated catalyst recyclability underscores the sustainability of this approach. To the best of our knowledge, this is the first instance of achieving room‐temperature, one‐pot, and high‐concentration conversion of xylose into equimolar yields of xylonic acid and xylitol without employing any external gas source.

## Results and Discussion

### Catalytic Performance of Commercial Metal (Pt, Rh, Ru, Pd)/C Catalysts

**Figure 1:**
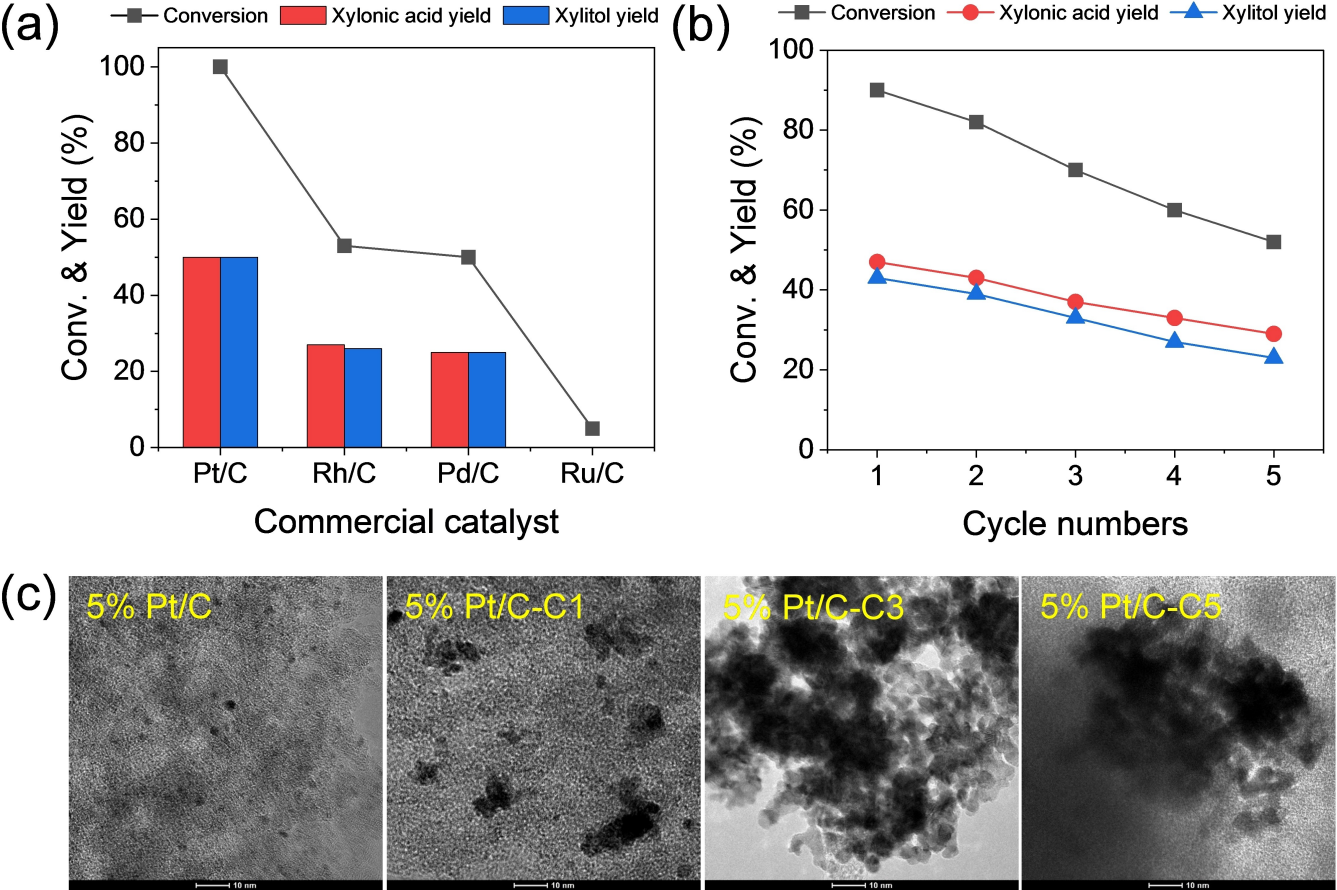
(a) Comparison of the catalytic performance of various commercial noble metal‐supported carbon catalysts and (b) catalytic recycling test of 5 % Pt/C. Reaction conditions: 2 M xylose, 2 M KOH, 0.4 g catalyst, 25 °C, 6 mL H_2_O, 1 h. (c) TEM images of Pt/C (fresh, cycle 1, cycle 3, and cycle 5). Scale bar=10 nm.

Initial experimental investigations involved the use of commercial metal‐supported carbon catalysts to discern the catalytic activity of diverse metals in the processes of xylose dehydrogenation and *in situ* hydrogenation, which aimed for the coproduction of xylonic acid and xylitol. As shown in Figure 1a, Pt exhibits superior activity, achieving complete conversion with a 50 % yield for both xylonic acid and xylitol. Relatively less conversions of 53 % and 50 % were achieved by the Rh and Pd catalysts, respectively, producing comparable proportions (25 %–27 %) of xylonic acid and xylitol. These results indicate that the Pt, Rh, and Pd surfaces efficiently use the *in situ* generated hydrogen, which stems from xylose dehydrogenation, for the hydrogenation of xylose to xylitol. In contrast, Ru/C gave less than 10 % xylose conversion, surprisingly neither to xylonic acid nor to xylitol as products. The findings revealed that xylose, rather than converting into acid and alcohol products, transforms into isomers and oligomers, as indicated by the orange color observed in the product solution. This result aligns with a reaction conducted entirely without a catalyst. According to prior research detailing the occurrence of isomerization and epimerization in alkaline solutions,[^40^, ^45^] the observed results indicate the formation of oligomers from various sugar monomers, thereby emphasizing the nonactivity of Ru in the dehydrogenation of xylose.

To assess the catalytic stability of Pt/C, recycling tests were conducted on Pt/C. Each reaction cycle, which lasted for 1 h, involved the washing, drying, and reuse of the spent catalyst for subsequent tests. In Figure 1b, a noticeable decrease in xylose conversion and product yields can be observed as the number of cycles increases. By the fifth cycle, an orange product solution was obtained, and the conversion dropped to 52 %. This decline indicates a decrease in the reaction rate for xylose dehydrogenation over the Pt surface. Transmission electron microscopy (TEM) analysis of the Pt/C catalyst before and after the reaction, as shown in Figures 1c and Figure S1, revealed significant aggregation of Pt nanoparticles after the first cycle, which contributed to the decreased dehydrogenation activity. Previous reports have shown that dehydrogenation and oxidation reactions are influenced by nanoparticle size.^[47]^ Therefore, the observed agglomerations of Pt nanoparticles in the Pt/C catalyst significantly impede the dehydrogenation rates, which results in the formation of oligomers and isomers through base‐catalyzed pathways, as shown in Figure S2. The perceived color transition from colorless to yellow in the reaction product, as shown in Figure S3, indicates that side reactions become dominant during the recycling test of the Pt/C catalyst. These initial experiments demonstrated the effectiveness of Pt, Rh, and Pd in the dehydrogenation and hydrogenation of xylose in an alkaline solution at room temperature, and Pt exhibited the highest catalytic activity. However, its catalytic stability was compromised by particle aggregation in a highly alkaline reaction environment (pH=14).

### Screening of the Support Material

**Figure 2:**
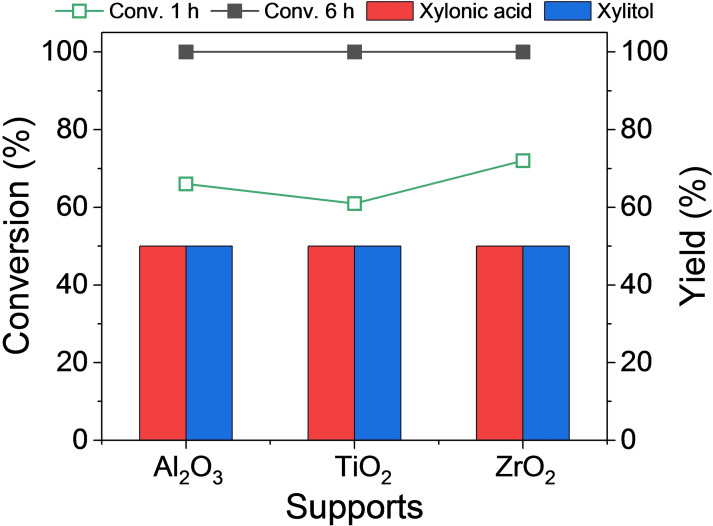
Comparison of the catalytic performance of the 5‐wt % Pt–supported metal oxides. Reaction conditions: 2 M xylose, 2 M KOH, 0.4 g catalyst, 25 °C, 6 mL H_2_O, 6 or 1 h (line graph for conversion, bar graph for yield).

Carbon materials possessing high surface area, good conductivity, and suitable chemical stability are commonly regarded as excellent supports for heterogeneous catalysis. However, the inherently chemically inert nature of carbon surfaces results in weak interactions between metals and carbon, leading to metal aggregation and sintering. To address these challenges and enhance the stability of heterogeneous catalysts, implementation of a robust strategy known as the strong metal–support interaction (SMSI) has emerged as a decisive approach. This strategy effectively tackles issues related to metal leaching and nanoparticle agglomeration.^[50]^ Consequently, 5 wt % Pt was impregnated on Al_2_O_3_, TiO_2_, and ZrO_2_, and the synthesized catalysts were used in the current reaction setup, as shown in Figure 2. Remarkably, xylose was completely converted into equivalent yields of xylonic acid and xylitol over all catalysts after a reaction time of 6 h, making the process of support screening challenging. Therefore, in the subsequent stage, the catalytic performance of the Pt catalysts with different metal oxide supports were compared based on their initial activities. After 1 h of reaction period, Pt/ZrO_2_ exhibited a superior reaction rate (87.6 h^−1^) for xylose conversion compared to Pt/γ‐Al_2_O_3_ (79.2 h^−1^) and Pt/TiO_2_ (73.2 h^−1^). In addition to reaction kinetics, the chemical stability of the support plays a pivotal role in the recyclability of the catalyst. In our previous study, we observed that amorphous γ‐Al_2_O_3_ transformed into crystalline boehmite under highly alkaline conditions.^[51]^ In addition, the Ti^4+^ ions in the TiO_2_ support can be partially reduced (Ti^4+^ to Ti^3+^) in the reducing environments.^[52]^ On the contrary, ZrO_2_ is an acid–base bifunctional metal oxide support, and the ZrO_2_ structure remains stable under both oxidizing and reducing atmospheres, making it an ideal support for high‐pH reactions.^[53]^ Therefore, in this study, ZrO_2_ was selected as the support material for Pt primarily because of its exceptional chemical stability in alkaline environments. Previous studies have substantiated the presence of SMSI between Pt and ZrO_2_, further reinforcing this choice.^[54]^ Consequently, Pt/ZrO_2_ catalysts were synthesized with varying Pt loading (1 %, 3 %, 5 %, and 9 %) (Figure S4), and their physicochemical properties were comprehensively characterized. This analysis aimed to elucidate the interplay between the physicochemical properties of Pt/ZrO_2_ and their impact on the catalytic activity and stability in the context of xylose dehydrogenation and *in situ* hydrogenation.

### Pt/ZrO_2_ Catalyst Characterizations

The physicochemical properties of ZrO_2_ and Pt/ZrO_2_ are detailed in Table 1. The textural properties, Pt loading, and Pt dispersion [based on the Pt quantity detected by inductively coupled plasma atomic emission spectroscopy (ICP‐AES)] of the catalysts were assessed using N_2_ isotherm, ICP‐AES, and CO chemisorption, respectively. The ZrO_2_ support′s surface area was determined to be 32.9 m^2^/g. Pt impregnation resulted in an insignificant difference in the surface area of the supported catalysts. The pore size of ZrO_2_ diminished from 34–27 nm as the Pt content increased, which is attributed to the incorporation of Pt nanoparticles within the interparticle spaces of ZrO_2_. ICP‐AES analysis confirmed that the prepared catalysts possess Pt amounts that matched with the Pt added during the preparation stage. The dispersion of metallic Pt, which was calculated through CO chemisorption, exhibited a linear decrease (from 72 %–47 %, 37 %, and finally 26 %) as the metal content increased from 1 %–9 %. This indicates an increase in the particle size of Pt.

**Table 1:** Textural properties of the Pt/ZrO_2_ catalysts.

Catalysts	Pore diameter (nm)^[a]^	Pore volume (cm^3^/g)^[a]^	Pt contents (%)^[b]^	Metal dispersion (%)^[c]^
ZrO_2_	34.0	0.28	‐	‐
1 % Pt/ZrO_2_	36.0	0.30	1.0	72
3 % Pt/ZrO_2_	32.0	0.27	2.9	47
5 % Pt/ZrO_2_	29.5	0.26	4.9	37
9 % Pt/ZrO_2_	27.3	0.25	8.8	26

[a] Analyzed using N_2_ isotherm, DFT method. [b] Determined using ICP‐AES. [c] Analyzed using CO chemisorption.

**Figure 3:**
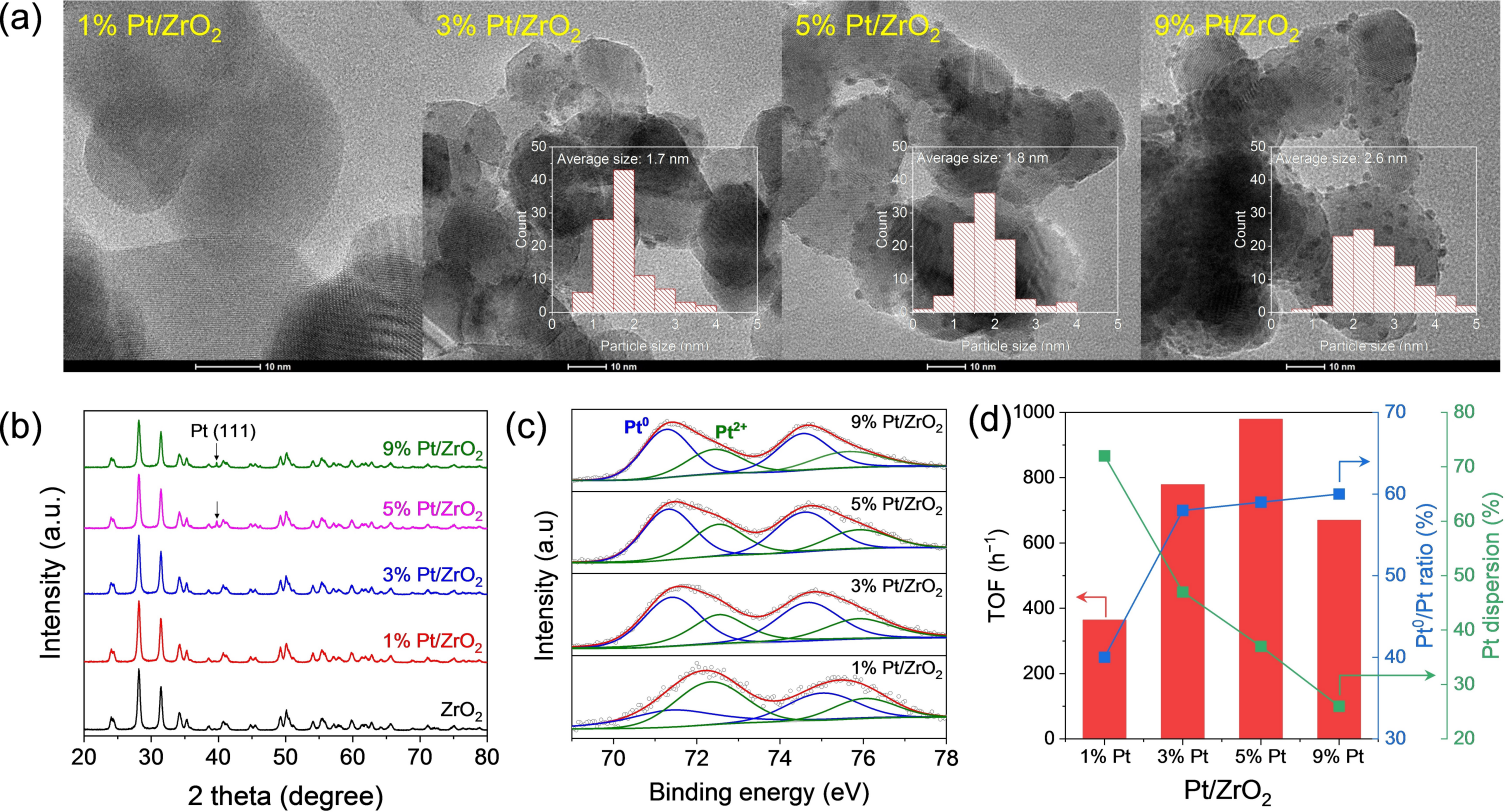
(a) TEM images, (b) PXRD patterns, and (c) Pt 4 f XPS results of the Pt/ZrO_2_ catalysts. (d) Correlation between the TOF and structural properties of the catalysts. Reaction conditions: 2 M xylose, 2 M KOH, 6 mL water, xylose/Pt ratio=120.

The high‐resolution TEM (HR‐TEM) images of the 1 %–9 % Pt/ZrO_2_ catalysts are shown in Figure 3a. In the case of 1 % Pt/ZrO_2_, Pt nanoparticles were not observed, indicating that Pt exists as single atoms or subnanosized clusters on the ZrO_2_ surface. The average particle sizes of 3 % Pt/ZrO_2_, 5 % Pt/ZrO_2_, and 9 % Pt/ZrO_2_ were 1.7, 1.8, and 2.6 nm, respectively, and the Pt nanoparticles were uniformly dispersed across the entirety of the ZrO_2_ support, as supported by the EDS map (Figure S5). The X‐ray diffraction (XRD) patterns of the Pt/ZrO_2_ catalysts, as presented in Figure 3b, show that all catalysts exhibited the characteristic peaks of monoclinic ZrO_2_. No metallic Pt peak was observed for the low‐Pt‐loading (1 % and 3 %) catalysts, and a small Pt (111) peak was observed for 5 % Pt/ZrO_2_ and 9 % Pt/ZrO_2_, indicating that the Pt nanoparticles are minute and well dispersed, corroborating the TEM and EDS analyses. X‐ray photoelectron spectroscopy (XPS) measurements were employed to define the chemical states of the Pt species in the Pt/ZrO_2_ catalysts, as shown in Figure 3c. The Pt 4 f XPS results indicate the copresence of Pt^0^ (centered at 71.0–71.3 eV and 73.3 eV for Pt 4f^7/2^ and Pt 4f^5/2^, respectively) and Pt^2+^ (centered at 72.2–72.4 eV and 74.7 eV for Pt 4f^7/2^ and Pt 4f^5/2^, respectively) in all catalysts. The Pt^0^/Pt^2+^ ratio in each catalyst was calculated from the peak area of the deconvoluted XPS data. In 1 % Pt/ZrO_2_, less than 40 % of Pt exists as Pt^0^ even after reduction because most of Pt has an SMSI with the ZrO_2_ support. This observation was also supported by the absence of Pt nanoparticles in 1 % Pt/ZrO_2_ as determined by TEM analysis. As the Pt loading increased, the Pt^0^ portion increased; 3 % Pt/ZrO_2_, 5 % Pt/ZrO_2_, and 9 % Pt/ZrO_2_ contained 58 %, 59 %, and 60 % of metallic Pt, respectively, but a large portion of Pt (40 %–60 %) still existed as Pt^2+^, indicating the presence of surface defects in the ZrO_2_ structure, which were responsible for the SMSI with the Pt species.

To compare the initial activities of the 1 %–9 % Pt/ZrO_2_ catalysts, a series of reactions was conducted for 15 min using 2 M xylose, 2 M KOH, and xylose/Pt=120. Figure 3d illustrates the correlation between the turnover frequency (TOF) of the catalyst and the Pt^0^/Pt ratio (from XPS analysis) and Pt dispersion (from CO chemisorption analysis). The obtained TOFs were 364.2, 779.3, 979.3, and 669.7 h^−1^ for 1 % Pt/ZrO_2_, 3 % Pt/ZrO_2_, 5 % Pt/ZrO_2_, and 9 % Pt/ZrO_2_, respectively. The heightened activities of the catalysts with high Pt loadings up to 5 % can be ascribed to the increased metallic Pt ratio, which serves as an active site for xylose dehydrogenation and hydrogenation. Notably, the oxidized Pt species do not directly contribute to the reaction; however, they account for the SMSI with the ZrO_2_ support. In the case of the 9 % Pt/ZrO_2_ catalyst, increased particle size resulted in the reduction in activity. Consequently, 5 % Pt/ZrO_2_ exhibited the best performance because of its high metallic Pt ratio and the high dispersion of Pt nanoparticles.

**Figure 4:**
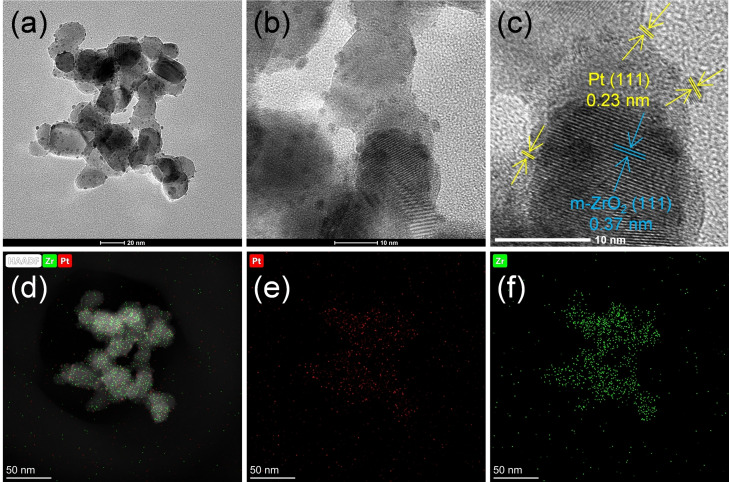
(a–c) HR‐TEM and (d–f) EDS mapping images of 5 % Pt/ZrO_2_.

The 5 % Pt/ZrO_2_ catalyst was further analyzed using HR‐TEM and EDS mapping, as illustrated in Figure 4. EDS mapping confirmed that the Pt particles are finely localized on the ZrO_2_ support and are well dispersed, which contribute to the high TOF. The high magnification of the TEM image (~10 nm) in Figure 4c distinctly displays the (111) lattice planes of the Pt nanoparticles with a *d* spacing of 0.23 nm, which is corroborated by XRD analysis.

### Pt/ZrO_2_ Catalyst Performance Evaluation

Several controlled experiments were conducted, as summarized in Table S1. In the absence of a catalyst under alkaline conditions, xylose was converted into various products, such as isomers and oligomers, and the solution turned pale yellow, with no formation of xylonic acid or xylitol (entry 1). Under neutral conditions, no xylose conversion was observed, even in the presence of the active Pt/ZrO_2_ catalyst (entry 2). Under acidic conditions (1 M H_2_SO_4_), more than 50 % of xylose was converted, but neither xylonic acid nor xylitol was produced (entry 3). These findings highlight the necessity of both a basic environment and a Pt catalyst for the initial dehydrogenation of xylose. In the presence of 10‐bar N_2_, xylose was fully converted into equal yields of xylonic acid and xylitol, which was identical to the reaction performed in the absence of any gas (entry 4). After the reaction, the N_2_ gas was collected and analyzed using GC, where only N_2_ was detected, with no discernible peaks corresponding to H_2_, as shown in Figure S6. Considering that the amount of N_2_ used (8 mmol, calculated using the ideal gas equation) is similar to the amount of xylose (12 mmol), the absence of a H_2_ peak in the GC analysis indicates that all hydrogen generated during the dehydrogenation of xylose was fully consumed in the subsequent hydrogenation of xylose to xylitol, achieving a 100 % hydrogen transfer efficiency. The absence of C−C cleaved products, such as lactic acid, acetic acid, or formic acid, was confirmed by NMR spectroscopy analysis, as shown in Figure S7. To investigate the potential role of water as a hydrogen source, a reaction was performed using D_2_O instead of H_2_O (entries 5 and 6). The results mirrored those of the H_2_O reaction, with 100 % conversion of xylose and equal yields of xylonic acid and xylitol. The reaction products were analyzed using NMR spectroscopy, as shown in Figures S7d and S7e, where identical superimposable spectra were observed. The absence of an additional peak for [1‐^2^H‐xylitol] excludes the possibility of water serving as the hydrogen source in this reaction.

**Figure 5:**
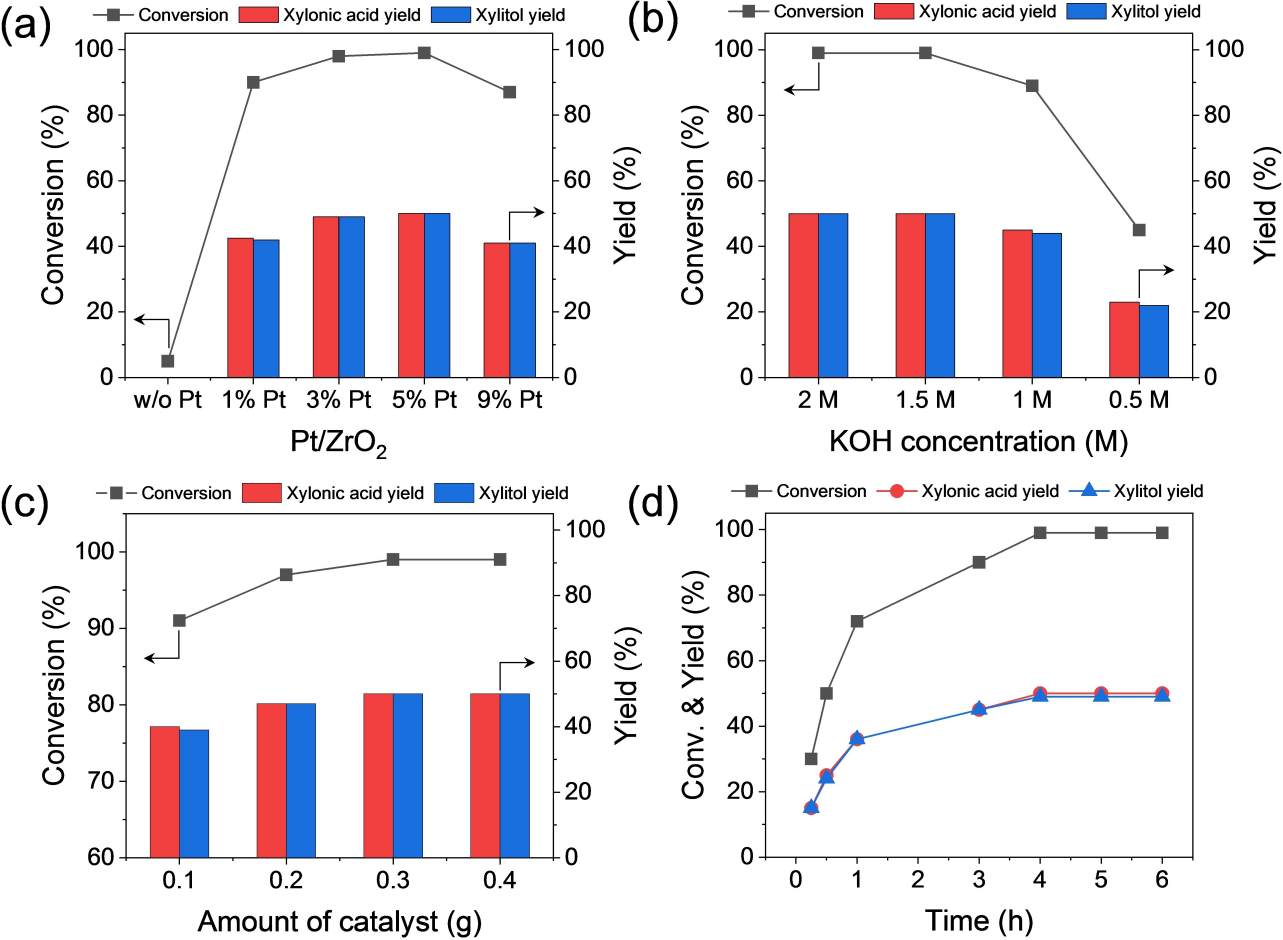
Xylose conversion and product yields as functions of various parameters. (a) Comparison of different Pt loadings (1 %–9 %), (b) effect of KOH concentration (0.5–2.0 M), (c) effect of catalyst amount (0.1–0.4 g), and (d) effect of reaction time (0–6 h). Standard reaction conditions: 2 M xylose, 2 M KOH, 0.4 g Pt/ZrO_2_, 25 °C, 6 mL H_2_O, 6 h, 1,400 rpm.

The catalytic performance of the Pt/ZrO_2_ catalysts was further assessed, as shown in Figure 5. The reaction was performed over a 6‐h reaction period using the 1 %–9 % Pt/ZrO_2_ catalysts while maintaining a Pt‐to‐xylose molar ratio of 1 : 120, as shown in Figure 5a. Notably, a negligible conversion was observed when the reaction was conducted using ZrO_2_ without Pt as the catalyst, with the formation of isomers and oligomers instead of xylonic acid and xylitol. The 5 % Pt/ZrO_2_ catalyst exhibited a 100 % conversion of xylose, with equivalent selectivity for xylonic acid and xylitol. Compared to this, lower conversions were achieved for 1 % Pt/ZrO_2_ (90 %), 3 % Pt/ZrO_2_ (98 %), and 9 % Pt/ZrO_2_ (87 %). Notably, despite the variations in conversion, the overall selectivity for both xylonic acid and xylitol remained consistent across all tested catalysts. This indicates that the hydrogen produced from the dehydrogenation of xylose to xylonic acid was promptly used for the hydrogenation of xylose to yield xylitol. Subsequently, a series of reactions was conducted under varying conditions, with the aim of optimizing the reaction parameters such as the KOH concentration and the reaction time, using the 5 % Pt/ZrO_2_ catalyst. With a fixed amount of xylose, the effect of varying quantities of base and catalyst was explored; then, the effect of reaction time was assessed based on the optimized amounts of base and catalyst. Bekkum et al. explored the kinetic impact of [OH^−^] concentration on the dehydrogenation of aldose.^[55]^ Their study revealed that the dehydrogenation rates of glucose increased with higher [OH^−^] concentrations, which was attributed to the accelerated formation of glucose anions. However, when the KOH concentration exceeded 2 M, the reaction rate declined due to the coadsorption of KOH on the catalyst surface.

Building upon these findings, our study used this reference to explore the influence of the base, as shown in Figure 5b. The results demonstrated that complete conversion of xylose was maintained when the KOH concentration was reduced from 2 M (equimolar to xylose) to 1.5 M. However, a notable decline in xylose conversion and product yields was observed with further reductions in KOH concentration, reaching 90 % conversion at 1 M and 45 % conversion at 0.5 M. The enhanced dehydrogenation rate of xylose at higher [OH^−^] concentrations is attributed to the accelerated formation of xylose anions. Conversely, lowering the KOH concentration from 1.5–0.5 M resulted in a decline in dehydrogenation rates due to diminished xylose deprotonation, particularly when the base concentration was less than half (0.5 and 1 M) that of xylose (2 M). Because hydrogen transfer efficiency is directly linked to dehydrogenation rate, the yields of both xylonic acid and xylitol decreased proportionally with lower KOH concentrations. This result aligns with the aforementioned published research and indicates that the base was not entirely consumed during the reaction. Notably, although the yields decreased linearly with the conversions, the selectivity for xylitol and xylonic acid remained constant (~50 %) regardless of the KOH concentration used. Our study aimed to optimize the reaction conditions to enhance the productivity of xylonic acid and xylitol; therefore, an equimolar amount of base (2 M) was employed for further investigation. Comprehensive examinations of aldose dehydrogenation kinetics, considering diverse quantities of aldose, base, and Pt, are available in the research conducted by Bekkum et al.^[55]^

In the next stage, the effect of catalyst amount was studied, as illustrated in Figure 5c. The results indicate that the mass of the catalyst also influenced the overall xylose conversions. The results remained consistent when using either 0.4 or 0.3 g of catalyst. However, a slight decrease in conversion from 100 %–97 % was observed upon reducing the catalyst amount to 0.2 g. Furthermore, a subsequent decline in conversion to approximately 90 % was noted when the catalyst amount was further reduced to 0.1 g. Again, the results confirmed that the dehydrogenation and hydrogenation selectivity remained constant regardless of the xylose conversion. Under alkaline conditions, xylose underwent dehydrogenation to form xylose anions catalyzed by the base. This serves as the initial step for the subsequent dehydrogenation, isomerization, and epimerization processes. The introduction of the Pt catalyst facilitates the dehydrogenation pathway, thereby promoting the selective production of xylonic acid. Importantly, the generated hydrogen promptly reacts to hydrogenate the remaining xylose to xylitol. Notably, the selectivity for both xylonic acid and xylitol remains consistently maintained at approximately 50 % irrespective of the variations in the reaction parameters. These results demonstrate that the proposed reaction setup is highly selective for the coproduction of xylonic acid and xylitol from xylose.

The effect of reaction time was investigated using 2 M xylose, 2 M KOH, and a 0.4 g Pt/ZrO_2_ catalyst, as shown in Figure 5d. During the first hour, the xylose conversion was kinetically fast, reaching 72 %, with equal selectivity for both xylitol and xylonic acid. A further increase in the reaction time resulted in gradually increased xylose conversion, with the system reaching saturation at 100 % conversion after 6 h. The yields of both products also increased linearly with the reaction time, confirming that the dehydrogenation and hydrogenation reactions proceed in parallel, thereby maintaining a consistent overall selectivity regardless of the reaction time.

**Figure 6:**
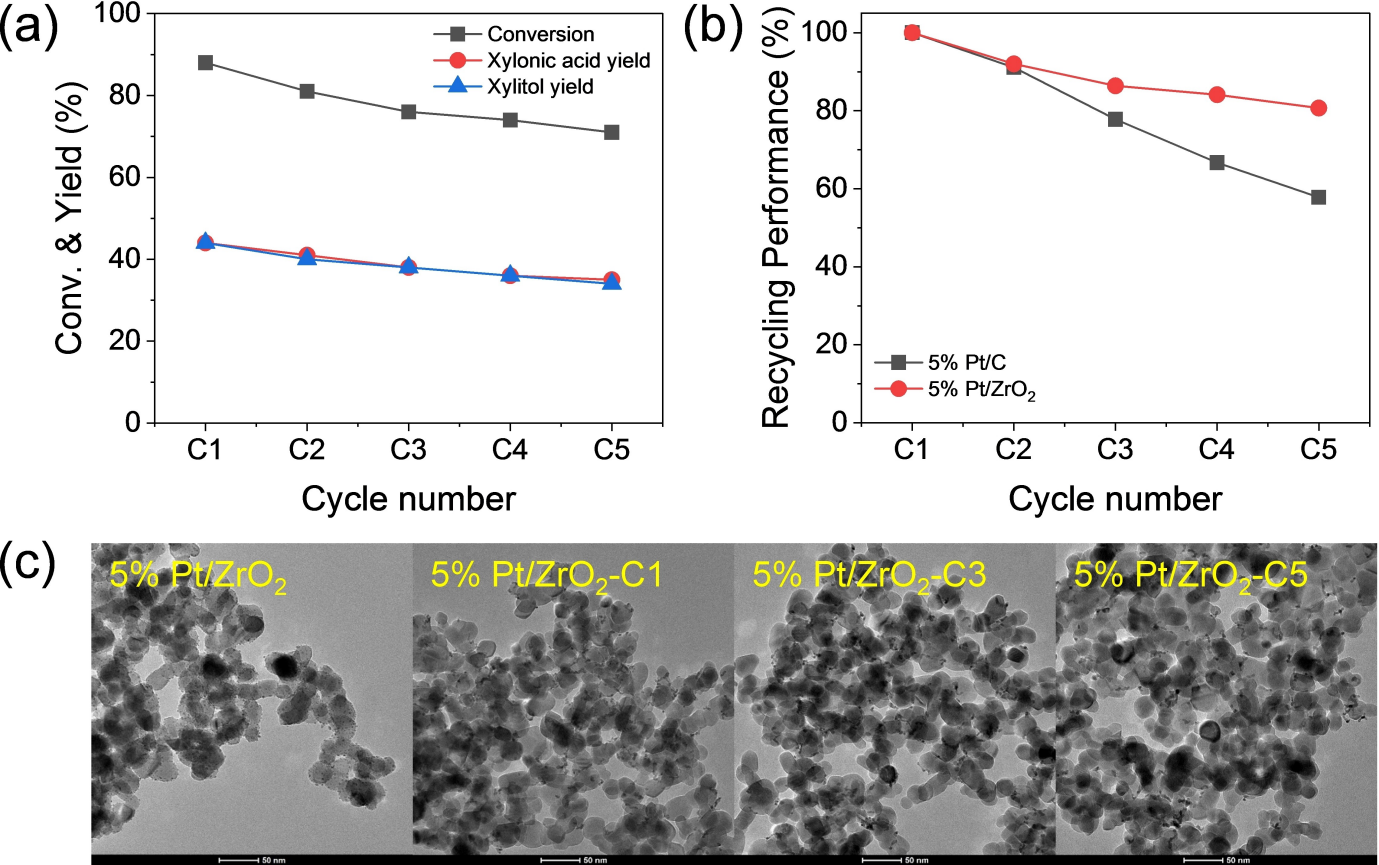
(a) Catalytic recycling test of 5 % Pt/ZrO_2_. (b) Recycling performance of 5 % Pt/ZrO_2_ and 5 % Pt/C. Reaction conditions: 2 M xylose, 2 M KOH, 0.4 g Pt/ZrO_2_, 25 °C, 6 mL H_2_O, 3 h. (c) TEM images of 5 % Pt/ZrO_2_ before and after the reactions (cycle 1, cycle 3, and cycle 5).

Then, catalytic recycling tests were conducted to investigate the stability and deactivation of the synthesized Pt/ZrO_2_ catalyst. Figure 6a shows the conversion and yields from five consecutive runs under the reaction parameters of 2 M xylose, 2 M KOH, and 0.4 g of 5 % Pt/ZrO_2_ catalyst. Each reaction was conducted for 3 h, after which the catalyst was removed from the reaction mixture, washed several times with water, dried overnight in an oven, and used in the subsequent run. Figure 6b shows a comparison of the recycling performance of commercial 5 % Pt/C and the synthesized 5 % Pt/ZrO_2_. The activity of 5 % Pt/ZrO_2_ decreased to 81 % after the fifth recycling test, while 5 % Pt/C showed a more significant reduction in activity, dropping to 58 %.

It has been reported that in sugar transformation reactions, the agglomerations of the active metal and the strong adsorption of organic compounds on the metal surface limit recyclability efficiency.^[56]^ To investigate the structural changes of the catalyst during the reaction, we characterized the spent catalyst using thermogravimetric analysis (TGA), XPS analysis, and TEM analysis, as shown in Figure 6c, Figures S8, S9, and S10. In our study, a slight weight loss observed in the TGA of the samples collected after the reactions might indicate the deposition of organic compounds, such as reactants, intermediates, or products (Figure S8). In addition, the average Pt particle size slightly increased after successive cycles, as seen in the TEM images, although this increase was less significant compared to that observed in Pt/C (Figure S3 versus Figure S9). We infer that the growth in active metal particle size, along with the deposition of organic compounds on the catalyst surface, may contribute to the decline in initial activity. Moreover, XPS analysis of the spent catalysts revealed a decrease in the binding energy of Pt after each reaction cycle due to the strong interaction between K^+^ ions and the Pt surface, resulting in an increased electron density of Pt, as illustrated in Figure S10. The presence of K^+^ ions cannot be completely excluded as a factor hindering xylose adsorption in the subsequent cycles. When comparing the stability of Pt/ZrO_2_ and Pt/C, the former exhibited superior recyclability, as shown in Figure 6b. This can be explained by their TEM images (Figures 6c and Figure S3), where less particle agglomerations were observed in Pt/ZrO_2_ compared to Pt/C. Moreover, in contrast to the orange color of the reaction sample after the fifth cycle for Pt/C, a transparent reaction sample was obtained after the fifth cycle for Pt/ZrO_2_ (Figure S11), indicating that the dehydrogenation rate over the Pt surface remains significantly faster than the side reactions catalyzed by the base.

### Extension of Sugar

**Figure 7:**
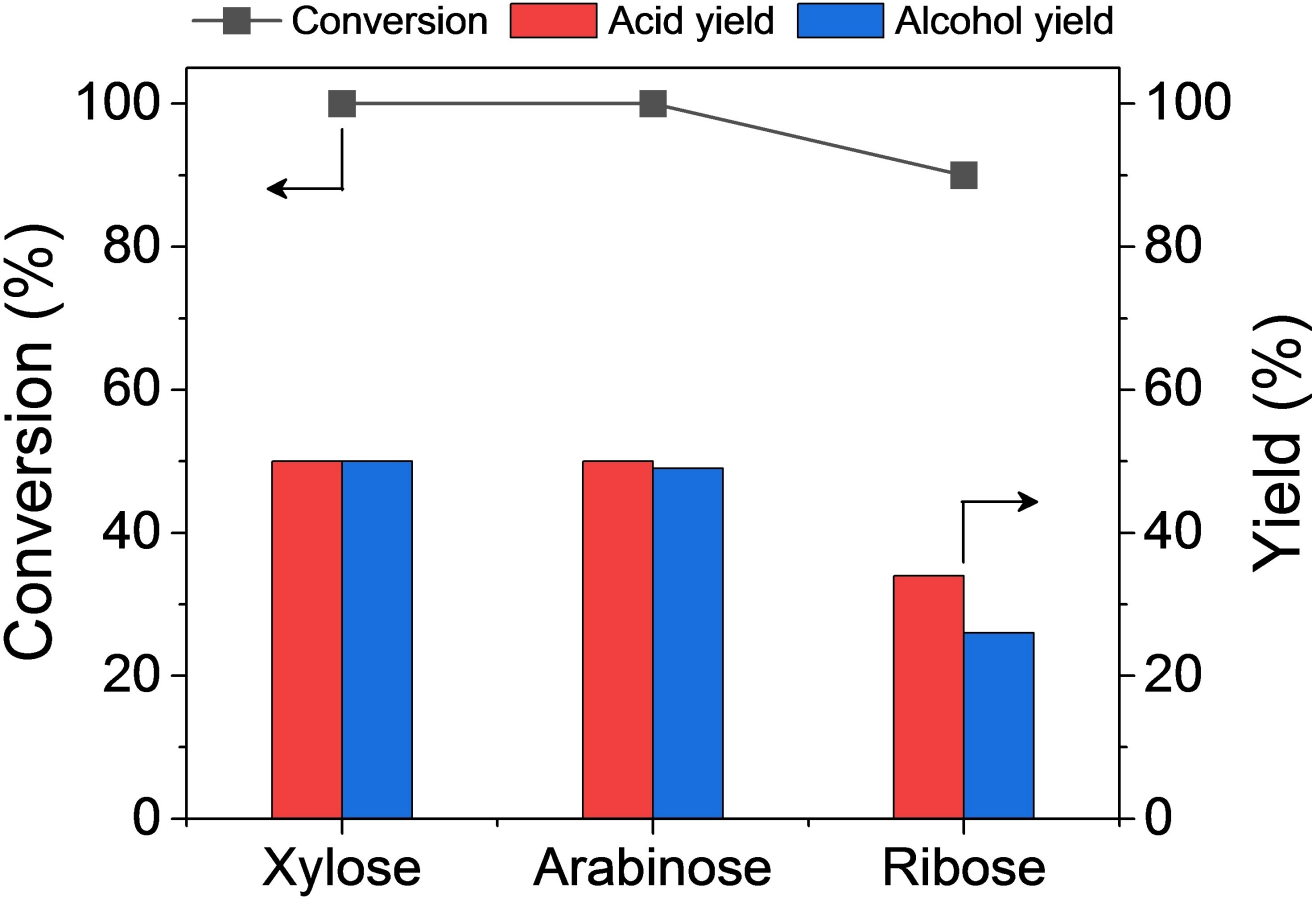
Pentose conversion results over Pt/ZrO_2_. Reaction conditions: 2 M pentose, 2 M KOH, 0.4 g 5 % Pt/ZrO_2_, 25 °C, 6 mL H_2_O, 6 h (line graph for conversion, bar graph for yields).

To expand the range of substrates, additional pentoses such as arabinose and ribose were employed as reactants, as shown in Figure 7. The results affirmed that akin to our prior research, these reducing sugars are readily transformed into their corresponding acid salts (arabinonic acid and ribonic acid) and alcohols (arabitol and ribitol). The overall activity of arabinose paralleled that of xylose; however, ribose yielded fewer conversions due to the structural difference.

### Reaction Pathways

**Scheme 2:**
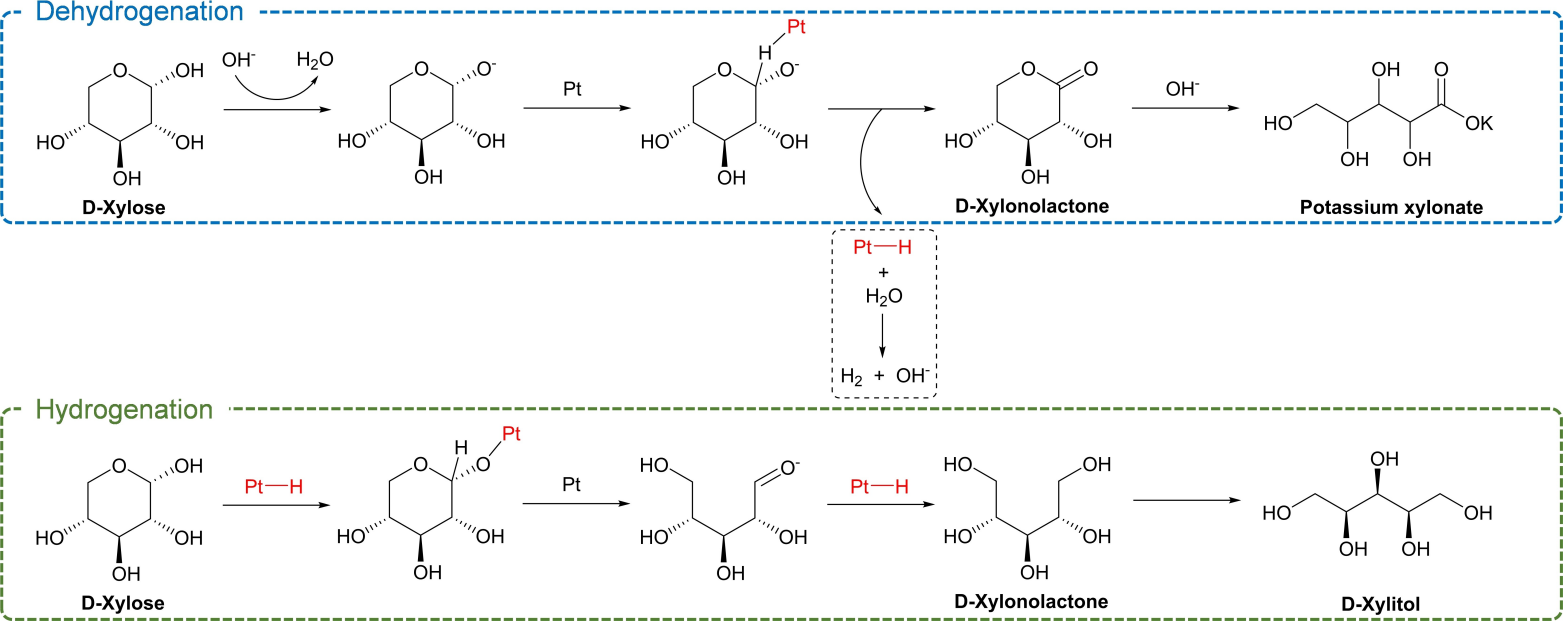
Plausible reaction mechanism for the conversion of xylose into xylonic acid and xylitol.

On the basis of the obtained reaction results, a plausible reaction mechanism for xylose conversion in the presence of the Pt catalyst and KOH is proposed in Scheme 2. The dehydrogenation and hydrogenation reactions follow distinct reaction pathways, with differing rate‐determining steps.^[58]^ Xylose deprotonation is catalyzed by the base, generating xylose anions. In the presence of a Pt catalyst, the dehydrogenation of xylose anions generates xylonolactone, which is considered the rate‐determining step in xylose dehydrogenation. The base further catalyzes the hydrolysis of xylonolactone to potassium xylonate. Simultaneously, xylose hydrogenation occurs through its adsorption on the Pt surface and the transfer of H to sugar to form a –OH group. Pentose could present as open‐chain aldehyde in acidic water or as pyranose in alkaline water, and the aldehyde group (–CHO) of the open‐chain structure can be hydrogenated to the corresponding sugar alcohol on the metal surface.^[59]^ The observed 50 : 50 selectivity for xylonic acid and xylitol, i. e., 100 % hydrogen transfer efficiency, under our reaction conditions is noteworthy considering that excess amounts of hydrogen source are generally used in the transfer hydrogenation reaction.

**Figure 8:**
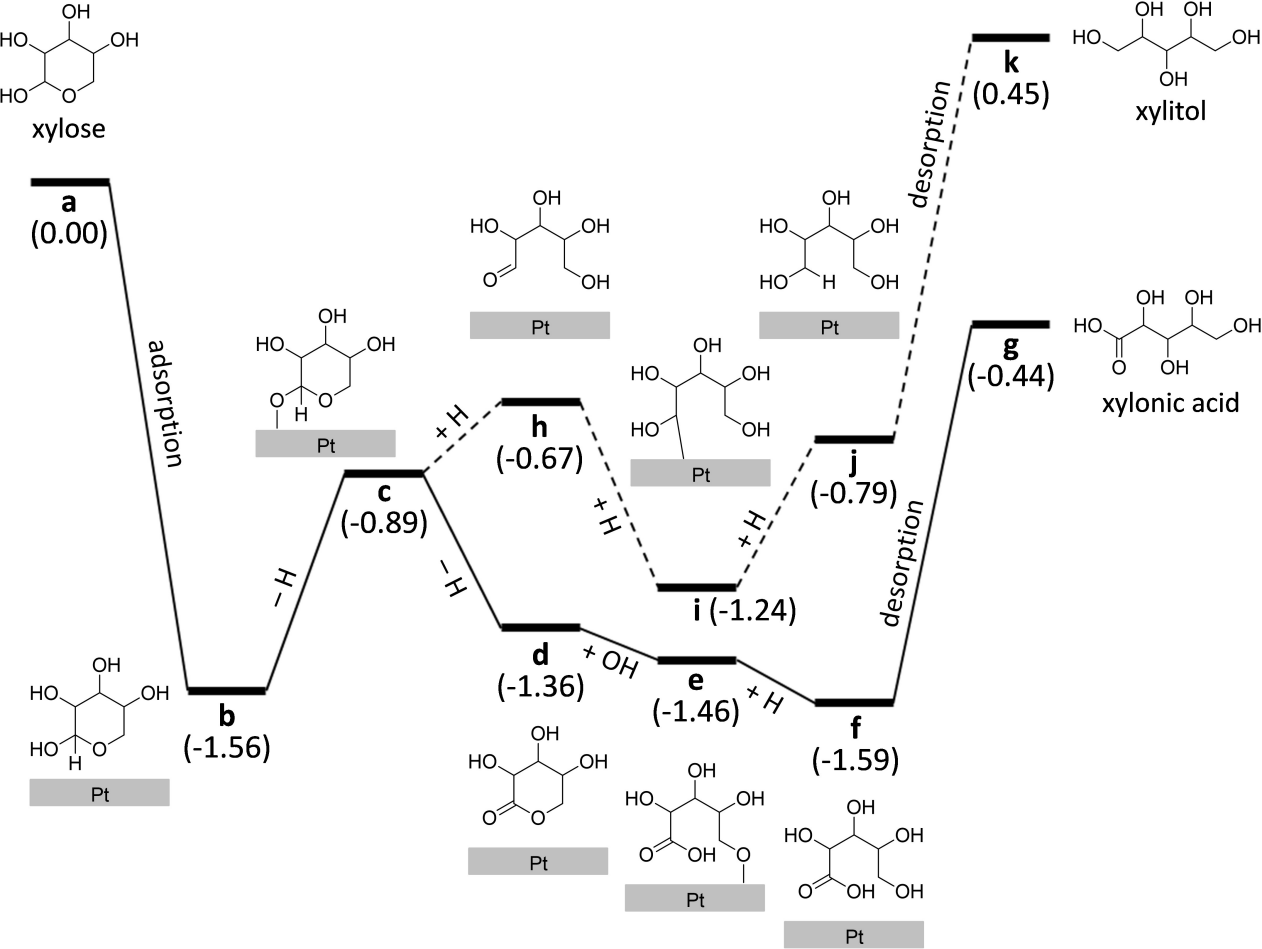
Reaction energy diagram (in electronvolts) for the pathways from xylose to xylonic acid (solid line) and to xylitol (dashed line).

First‐principles calculations were performed to elucidate the reaction energy profile for pathways leading to the production of xylonic acid and xylitol from xylose, which were simultaneously achieved using a Pt nanocatalyst impregnated on a ZrO_2_ support. To elucidate the reduced aggregation of the Pt nanocatalyst on ZrO_2_ in contrast to a carbon support during repeated catalytic reactions, the interfacial interaction between Pt and the support materials was investigated using a model system, i. e., a Pt_10_ cluster adsorbed on ZrO_2_ (111) and graphite surfaces (Figure S12). The adsorption energy of the Pt_10_ cluster on ZrO_2_ (111) was determined to be −6.12 eV, which is significantly stronger than that on graphite (−1.52 eV) by 4.6 eV. This result supports our experimental results that the aggregation of Pt nanoparticles was prevented over a larger number of catalytic cycles using the ZrO_2_ support compared to the carbon support (Figure 6b), highlighting the importance of creating an SMSI with the Pt nanocatalysts for long‐term stability. Figure 8 presents the reaction energy profiles constructed for the proposed reaction mechanisms, starting from xylose (a) to xylonic acid (g) and to xylitol (k) (Figures S13 and S14 for the energy changes of each reaction step, Figure S15 for the carbon numbers). The dehydrogenated form of xylose (c) could serve as an initial reactant in the Pt catalyst considering basic experimental conditions. The computational results indicate that the dehydrogenation of c (c→d, ΔE=−0.46 eV) to produce xylonic acid is more favorable than its hydrogenation (c→h, ΔE=+0.22 eV) to produce xylitol. Consequently, the overall reactions yielding xylonic acid and xylitol are exothermic (ΔE=−0.44 eV) and endothermic (ΔE=+0.45 eV), respectively, with respect to the energy of isolated xylose (a). However, for both pathways, the reaction step that demands the highest energy is the desorption of the produced chemical species from the Pt surface. The evaluated desorption energies for xylonic acid and xylitol are comparable to each other: ΔE=+1.24 eV (j→k) and ΔE=+1.15 eV (f→g), respectively. In contrast, while the product desorption steps are highly endothermic, the energy changes for the hydrogenation of xylose (c→h, ΔE=+0.22 eV; h→i, ΔE=−0.57 eV; i→j, ΔE=+0.45 eV) are much lower. Furthermore, considering the presence of *in situ* generated Pt−H species that were not considered in our calculation, this result supports the obtained 50 : 50 selectivity for xylonic acid and xylitol. In addition, the higher desorption energy in comparison to any intermediate reaction step indicates the feasibility of reversible reactions on the Pt surface.

**Figure 9:**
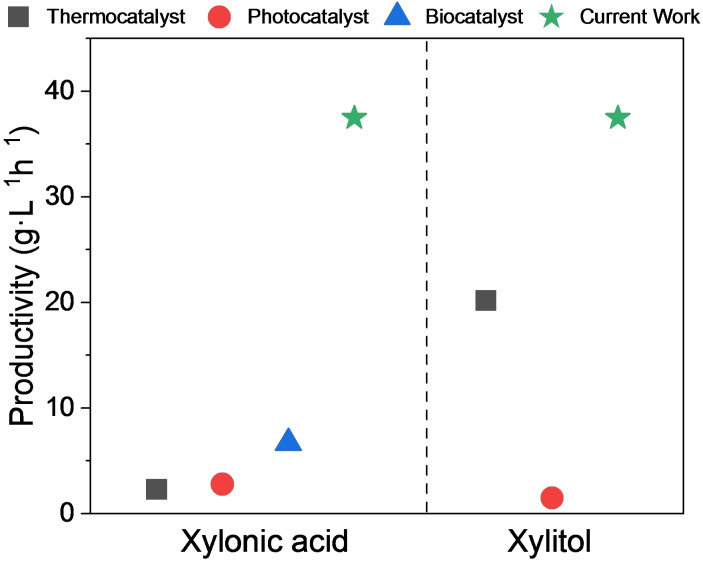
Comparison of the productivities of various catalytic processes for the coproduction of xylonic acid and xylitol.[^25^, ^60^]

The current methods for synthesizing xylonic acid and xylitol include biological and chemical methods, each associated with pros and cons. A key descriptor often used to compare the effectiveness of these processes is the calculation of volumetric productivity, where it mainly depends on the initial substrate concentration and the overall yield of the product with time. Herein, the thermocatalytic, photocatalytic, and biocatalytic productivities were compared with our current work, as shown in Figure 9. The overall productivity of xylonic acid and xylitol in the current reaction system is six times higher than those of others and stands out among all other reported processes.

The coproduction of highly concentrated biomass‐derived value‐added products at room temperature emphasizes the sustainability of this process. Efficient separation between the resulting sugar acid and sugar alcohol is a critical step currently being addressed by our group using bipolar membrane electrodialysis, alongside process optimization through simulation. Investigating reaction kinetics and controlling product selectivity through the development of tailored catalysts, such as bimetal catalysts or alternative supports, will be key to further improving process efficiency and understanding. In addition, exploring continuous‐flow reactions without external gases presents an exciting opportunity to enhance the overall process performance

## Conclusions

Here, we have proposed a novel one‐pot reaction for the coproduction of xylonic acid and xylitol from xylose via transfer hydrogenation, which was executed at ambient temperature in the presence of a Pt catalyst and a base, without the need for external gases. Among the various metal‐supported carbon catalysts investigated (Pt, Rh, Pd, or Ru), Pt/C exhibited superior catalytic performance, achieving complete conversion with a 50 % yield of both xylonic and xylitol. However, Pt/C suffered from a substantial decrease in activity during the recycling tests, which is attributed to significant sintering of the Pt nanoparticles. To address this challenge, Pt/ZrO_2_ catalysts with varying Pt loading (1 %, 3 %, 5 %, and 9 %) were synthesized, and their structural properties were meticulously characterized. Compared to Pt/C, the 5 % Pt/ZrO_2_ catalyst demonstrated remarkable stability during the recycling tests, which is attributed to the SMSI between Pt and the ZrO_2_ support, as supported by the computational result. The equal 50 % formation of both xylonic acid and xylitol observed in our experiment could be attributed to the well‐balanced neutral desorption energies of these products on the Pt surface compared to any intermediate step according to the computational results. This study paves the way for green research into the simultaneous production of concentrated sugar‐derived acids and alcohols at room temperature, using a single catalyst, without the need for any external gas.

## Experimental Section

### Chemicals

D‐(+)‐xylose (>99.5 %), D‐xylonic acid lithium salt (>99 %), D‐xylitol (>98 %), D‐(+)‐ribose (>99 %), D‐(+)‐arabinose, potassium hydroxide (KOH, 90 %), 5 % Pt/C, 5 % Rh/C, 5 % Ru/C, 5 % Pd/C, chloroplatinic acid hexahydrate (>37.5 % Pt basis), gamma alumina, and titanium (IV) oxide rutile nanopowder (particle size of <100 nm) were purchased from Sigma‐Aldrich. Glacial acetic acid and high‐performance liquid chromatography (HPLC) water were obtained from Samchun Pure Chemicals (Korea). All chemicals were used without further purification.

### Syntheses of the Catalysts

Pt/ZrO_2_ catalysts with varying Pt composition, ranging from 1 % to 9 %, were prepared using a simple wet impregnation method. The process began with the calcination of Zr(OH)_4_ powder (procured from MEL Chemicals) at a temperature of 600 °C to obtain monoclinic ZrO_2_. After this, a solution of chloroplatinic acid hexahydrate (H_2_PtCl_6_⋅6H_2_O) in deionized water was gradually introduced to a slurry of ZrO_2_ powder also in deionized water, all the while stirring continuously. The resulting mixture was left to stir for a duration of 6 h at room temperature, after which the solids were separated by evaporating the water and dried overnight in an oven at 100 °C. The powder was then calcined in a box furnace at 500 °C for a period of 4 h under a consistent airflow of 150 mL/min. Once cooled to room temperature, the calcined catalyst was further reduced at 300 °C for 2 h under a 5 % H_2_/Ar flow of 150 mL/min. To investigate the impact of the various supports, Pt was impregnated on TiO_2_ and Al_2_O_3_ using the same method applied for Pt/ZrO_2_.

### Catalytic Dehydrogenation and Hydrogenation of Xylose

The conversion of pentose was conducted in a microvial reactor with a capacity of 10 mL. Specific quantities of pentose (such as xylose, arabinose, or ribose), KOH, and the catalyst were integrated into 6 mL of water. This mixture was then agitated using a magnetic stirrer at a rate of 1,400 rpm for the desired reaction time at a temperature of 25 °C.

### Product Analysis

In all reactions, the product solution underwent vacuum filtration to isolate the catalyst. Subsequently, the sample was diluted with an ample amount of water (~10 times), thoroughly mixed to achieve a transparent sample, and refiltered using a 0.2 μm syringe filter. The resultant liquid product was subjected to HPLC analysis using an HPLC system (Youngin, YL9100) outfitted with a dual UV–vis detector at 218 nm and a refractive index detector. Organic products were distinguished in a Shodex Sugar SH1101 column, employing 10‐mM acetic acid as a mobile phase at a flow rate of 0.5 mL/min and maintaining a column temperature of 30 °C. The HPLC system was adjusted using a multipoint calibration method. Calculations for pentose conversions, product yields, and selectivity were based on the following formulas, as shown in **Figures** **S16** and **S17**.

(1) Conversion(%)=(Initialmolesofreactant-Finalmolesofreactant)/(Initialmolesofreactant)×100

(2) Yield(%)=(Molesofproduct)/(Molesofreactant)×100

(3) Selectivity(%)=Yield/Conversion×100

### Characterizations

The powder XRD (PXRD) patterns of the catalysts were obtained using an X‐ray diffractometer (Rigaku D/Max‐2200 V) emitting Ni‐filtered Cu Kα radiation (40 kV, 40 mA, λ=1.5406 Å) with a scan rate of 5 °C/min. The N_2_ adsorption–desorption isotherms were measured at 77 K using a Micromeritics Tristar 3,000. Prior to the analysis, the samples were activated at 200 °C for 2 h under vacuum. The specific surface area was determined via the Brunauer–Emmett–Teller method. TGA was performed using a thermal analyzer (Scinco, TGA−N 1,000), where the samples were heated at a rate of 5 °C/min from 25 °C to 800 °C under a constant flow of air set at 30 mL/min. The distribution of metal particles within the reduced catalysts was examined through TEM analysis, with a minimum of 200 metal particles taken into consideration for the calculation of the average metal particle size. TEM‐Talos (F200X system) operating at an accelerating voltage of 300 kV was used for this purpose. The XPS spectra were recorded on a Kratos AXIS SUPRA instrument (UK) with a monochromatic Al Kα X‐ray source operated at a pass energy of 20 eV. ICP‐AES was conducted on an ICAP 6,500 duo series analyzer (Thermo, USA). The dispersion of metal within the catalysts was assessed through CO pulse chemisorption conducted at 40 °C using 10 % CO/He gas by a Micromeritics Autochem II 2,920 V5.02. Prior to the experiment, the catalyst was activated under a helium flow (50 mL/min) at 250 °C for 1 h and subsequently reduced by 10 % H_2_/Ar gas (50 mL/min) at 250 °C for an additional hour.

### Computational Details

First‐principles calculations were performed in a manner of spin polarization using the FHI‐aims software package.^[64]^ The exchange–correlation energy was evaluated using the Perdew–Burke–Ernzerhof^[65]^ functional and the light Tier 1 numeric atom‐centered basis set. The scalar relativistic effect was considered with the atomic variant of zeroth‐order regular approximation,^[64]^ and the long‐range van der Waals interaction was corrected based on Hirshfeld partitioning of electron density.^[66]^ Ionic relaxations were performed until the atomic forces were below 0.02 eV/Å. Pt (111) as a catalytic surface was modeled with (5×5) supercells, and Γ‐centered 2×2×1 k‐point grids were used for the first Brillouin zone sampling. The slab models consist of five Pt layers, with the two bottom layers fixed in their bulk positions during ionic relaxations. The periodically replicated slabs were separated by ~20 Å of vacuum, and dipole correction was applied to prevent interactions between periodic slab images. Furthermore, to investigate the interfacial interaction between the Pt catalyst and support materials, we employed the tetrahedral Pt_10_ cluster^[67]^ as a model catalyst and monoclinic ZrO_2_ (111) and graphite as support materials. The (2×2) and (5×5) supercells of ZrO_2_ (111) and graphite, respectively, and Γ‐centered 2×2×1 k‐point grids were used for the first Brillouin zone sampling. The slab models consist of four layers for both systems, with the two bottom layers fixed in their bulk positions during ionic relaxations.

## Conflict of Interests

The authors declare no conflict of interest.

1

## Supporting information

As a service to our authors and readers, this journal provides supporting information supplied by the authors. Such materials are peer reviewed and may be re‐organized for online delivery, but are not copy‐edited or typeset. Technical support issues arising from supporting information (other than missing files) should be addressed to the authors.

Supporting Information

## Data Availability

Research data are not shared.
