# Supplementary material for: Simultaneous Coproduction of Xylonic Acid and Xylitol: Leveraging In Situ Hydrogen Generation and Utilization from Xylose
Source: ChemSusChem. 2024 Dec 27;18(5):e202401651. doi: 10.1002/cssc.202401651 (PMC11874631; doi:10.1002/cssc.202401651)
Supplement: Supplementary file 1 — Supporting Information [file CSSC-18-e202401651-s001.pdf]

ChemSusChem

Supporting Information

Simultaneous Coproduction of Xylonic Acid and Xylitol: Leveraging *In Situ* Hydrogen Generation and Utilization from Xylose

Ali Awad, Anil H. Valekar, Kyung-Ryul Oh, Fajar Prihatno, Jaehoon Jung,
Ajaysing S. Nimbalkar, Pravin P. Upare, Ji Hoon Kim, and Young Kyu Hwang*

Supporting Information

Simultaneous Coproduction of Xylonic acid and Xylitol: Leveraging In-situ Hydrogen Generation and Utilization from Xylose

Ali Awad[1, 2], Anil H. Valekar[1], Kyung-Ryul Oh[1], Fajar Prihatno[3], Jaehoon Jung[3], Ajaysing S. Nimbalkar[1, 2], Pravin P. Upare[4], Ji Hoon Kim[5], Young Kyu Hwang[1, 2]*

[1] Green Carbon Research Center, Korea Research Institute of Chemical Technology (KRICT), Daejeon 34114, Republic of Korea

[2] Department of Advanced Materials and Chemical Engineering, University of Science and Technology (UST), Daejeon 34113, Republic of Korea

[3] Department of Chemistry, University of Ulsan, Ulsan 44776, Republic of Korea

[4] R&D Center, ACTIVON Co., Ochang-eup, Cheongju 28104, Republic of Korea

[5]Chemical Process Solution Research Center, Korea Research Institute of Chemical Technology (KRICT), Daejeon 34114, Republic of Korea

* Corresponding author: **Young Kyu Hwang**

Email: ykhwang@krict.re.kr

List of Figures

Figure S1. TEM images and EDS maps of Pt/C before reaction and after reactions (cycle 1, cycle 3, and cycle 5). Scale bar = 50 nm.

Figure S2. Isomerization and epimerization pathways for D-xylose.

Figure S3. Photograph of reaction product solutions after cycle 1 and cycle 5 using commercial Pt/C catalyst.

Figure S4. Schematic illustration of 1–9% Pt/ZrO_2 synthesis procedure and photographs of the synthesized catalysts.

Figure S5. TEM images, particle size distributions, and EDS maps of 1–9% Pt/ZrO_2 catalysts. Scale bar = 50 nm and 10 nm.

Figure S6. GC chromatograms for the mixed H_2/N_2 gas (reference) and gas collected after reaction (product).

Figure S7. NMR spectra of (a) xylose, (b) xylonic acid, (c) xylitol, and (d) reaction product with H_2O as a solvent, and (e) reaction product with D_2O as a solvent (2 M xylose, 2 M KOH, 0.4 g Pt/ZrO_2, 25 °C, 6 mL H_2O).

Figure S8. TGA results of 5%Pt/ZrO_2 before reaction and after reactions (cycle 1, cycle 3, and cycle 5).

Figure S9. TEM images and EDS maps of 5%Pt/ZrO_2 before reaction and after reactions (cycle 1, cycle 3, and cycle 5). Scale bar = 50 nm and 10 nm.

Figure S10. Pt 4f XPS spectra of 5% Pt/ZrO_2 before reaction and after reactions (cycle 1, cycle 3, and cycle 5).

Figure S11. Photograph of reaction product solutions after cycle 1 and cycle 5 using 5% Pt/ZrO_2 catalyst.

Figure S12. Top and side views of optimized adsorption structures of Pt_{10} (a and c) on monoclinic ZrO_2(111) and (b and d) on graphite. For the top view images, only the first support layer is depicted for clarity. The interfacial close contacts, which are estimated using the scaled sum of van der Waals radii, are indicated with dashed lines.

Figure S13. The energy changes for each reaction step from xylose to xylonic acid (see **Figure 8** in main text). For D-xylose, α form is more stable than β by 0.07 eV as reported in previous study[S5]. H or OH species in parentheses, which is attached or removed from the molecule during reaction step, is additionally adsorbed on Pt(111) surface.

Figure S14. The energy changes for each reaction step from xylose to xylitol (see **Figure 8** in main text). For D-xylose, α form is more stable than β by 0.07 eV as reported in previous study[S5]. H or OH species in parentheses, which is attached or removed from the molecule during reaction step, is additionally adsorbed on Pt(111) surface.

Figure S15. Xylose structures with carbon numbers for better understanding on adsorption on Pt surface.

Figure S16. HPLC curves for a) xylose, b) lithium xylonate, c) xylitol standards, and d) reaction product at 100% conversion. A small peak at 20 min is from the mobile phase.

Figure S17. Calibration curves for a) xylose, b) lithium xylonate, and c) xylitol.

List of Tables

Table S1. Xylose conversion over various reaction conditions.

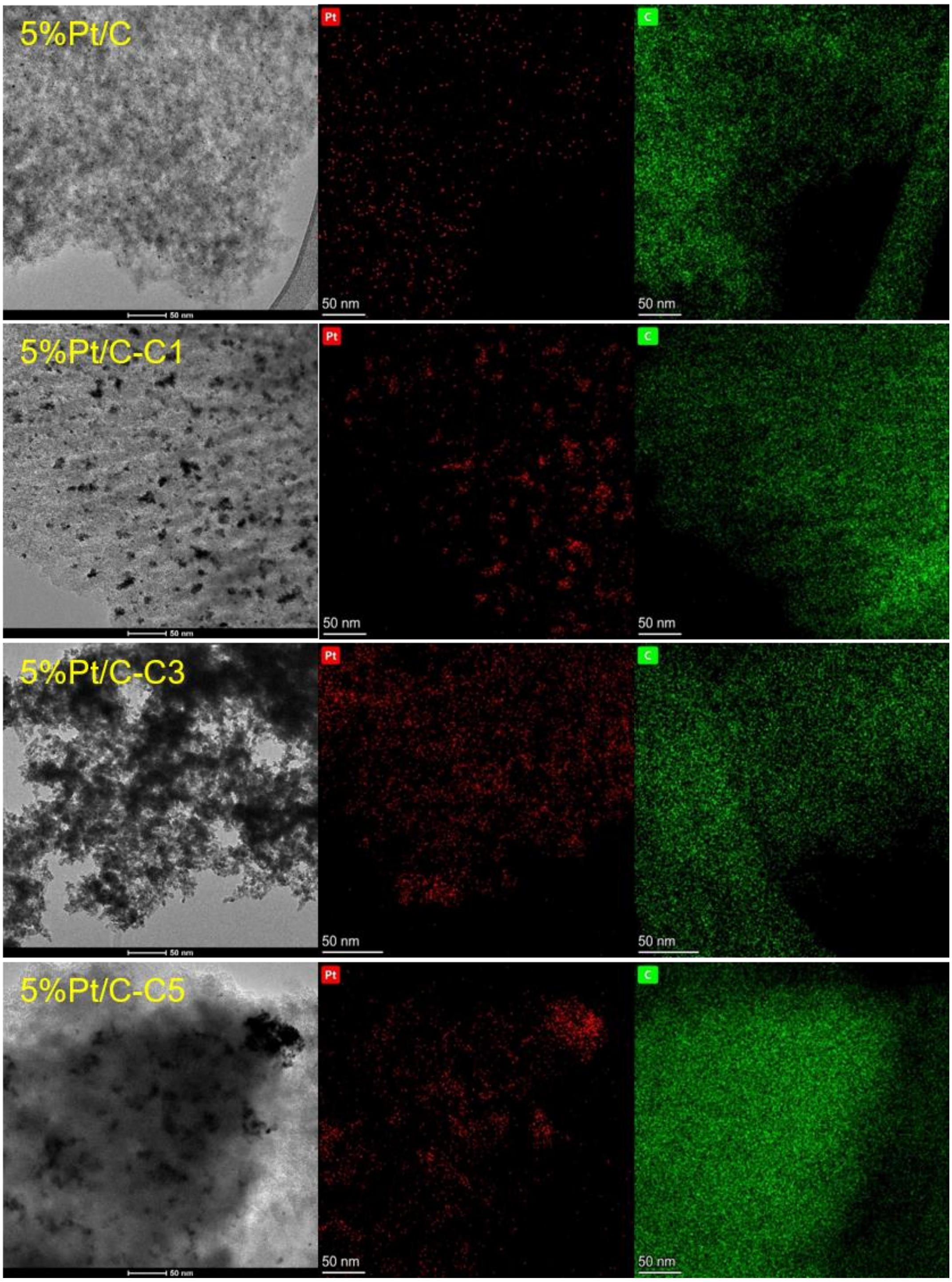

Figure S1. TEM images and EDS maps of Pt/C before reaction and after reactions (cycle 1, cycle 3, and cycle 5). Scale bar = 50 nm.

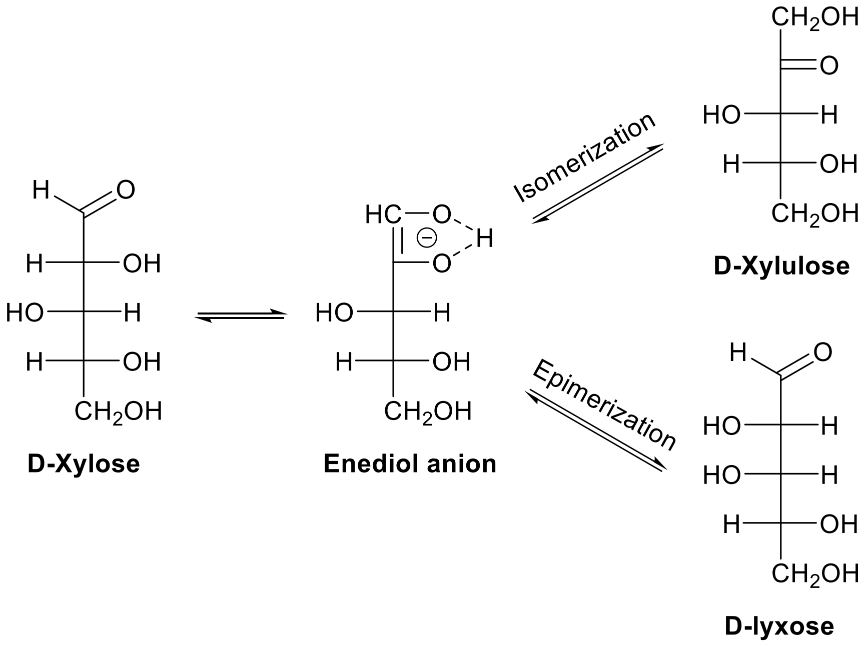

Figure S2. Isomerization and epimerization pathways for D-xylose.

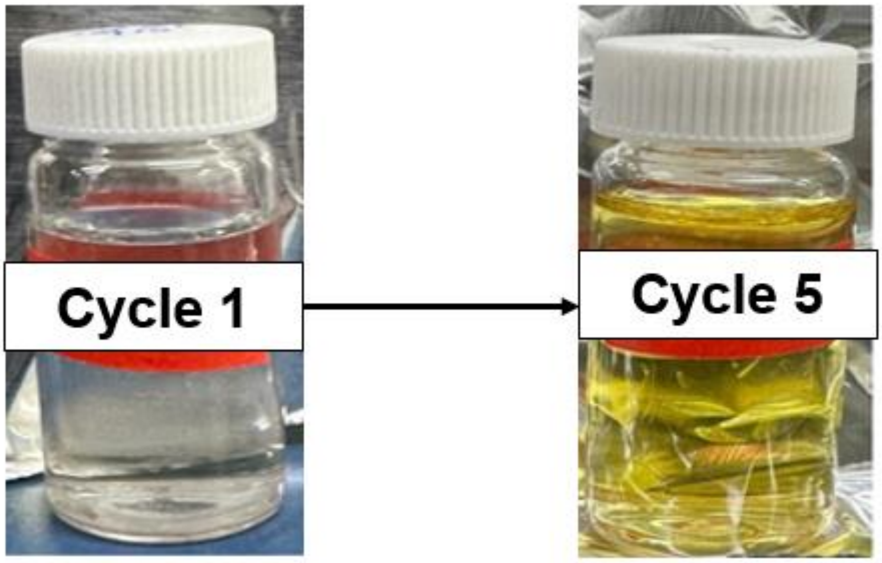

Figure S3. Photograph of reaction product solutions after cycle 1 and cycle 5 using commercial Pt/C catalyst.

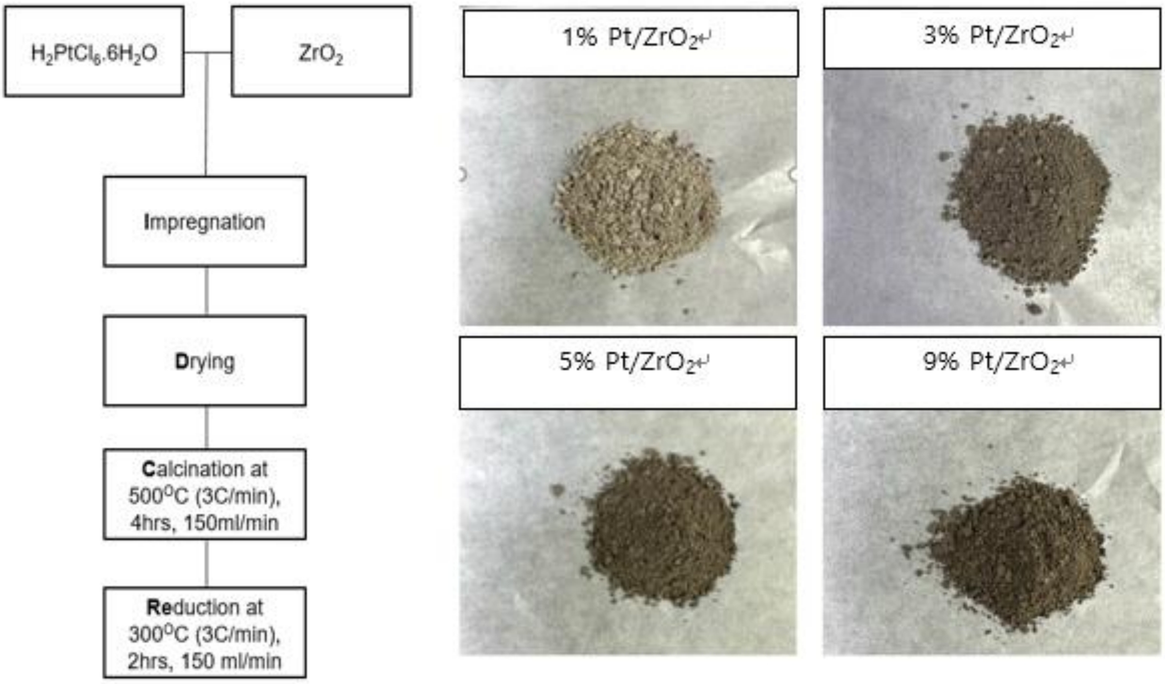

Figure S4. Schematic illustration of 1–9% Pt/ZrO_2 synthesis procedure and photographs of the synthesized catalysts.

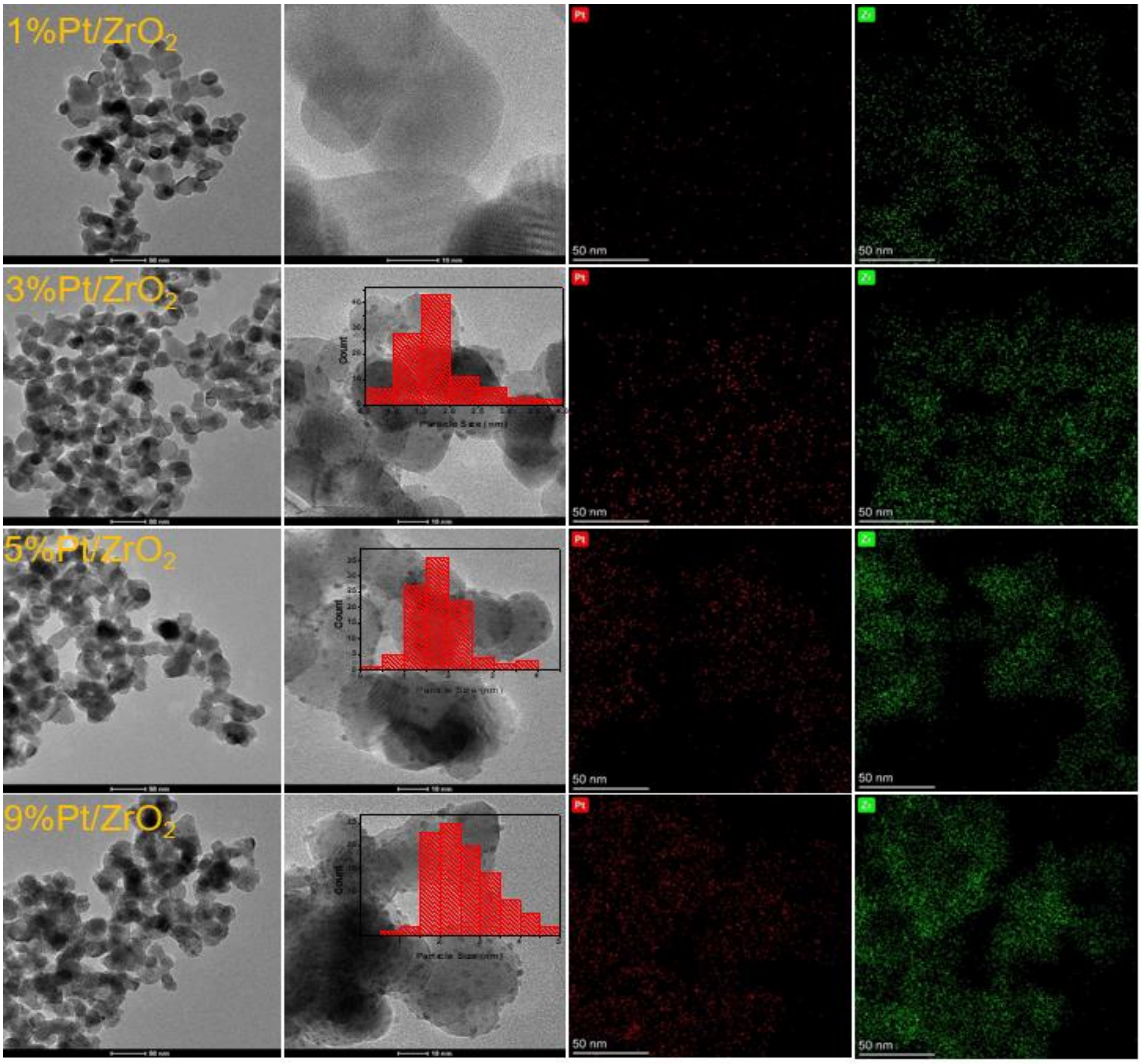

Figure S5. TEM images, particle size distributions, and EDS maps of 1–9% Pt/ZrO_2 catalysts. Scale bar = 50 nm and 10 nm.

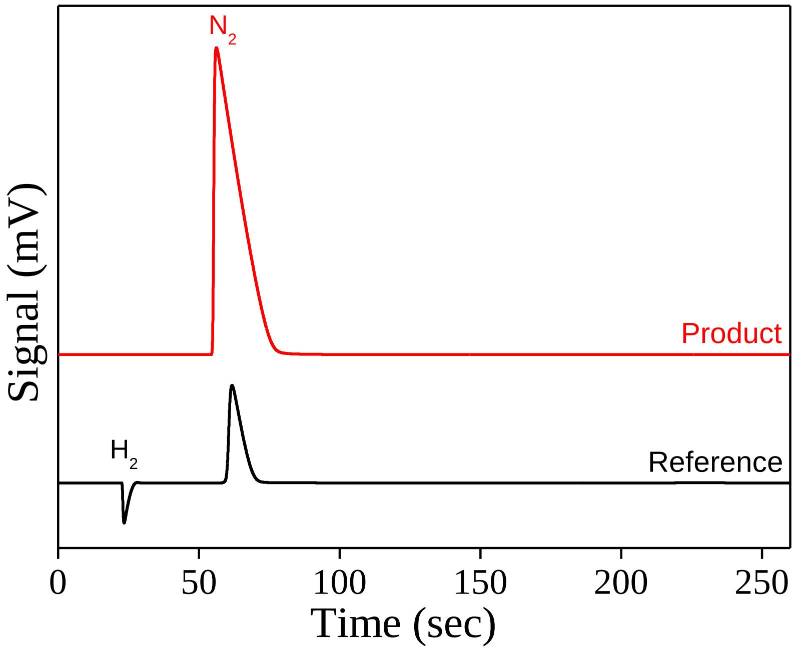

Figure S6. GC chromatograms for the mixed H_2/N_2 gas (reference) and gas collected after reaction (product).

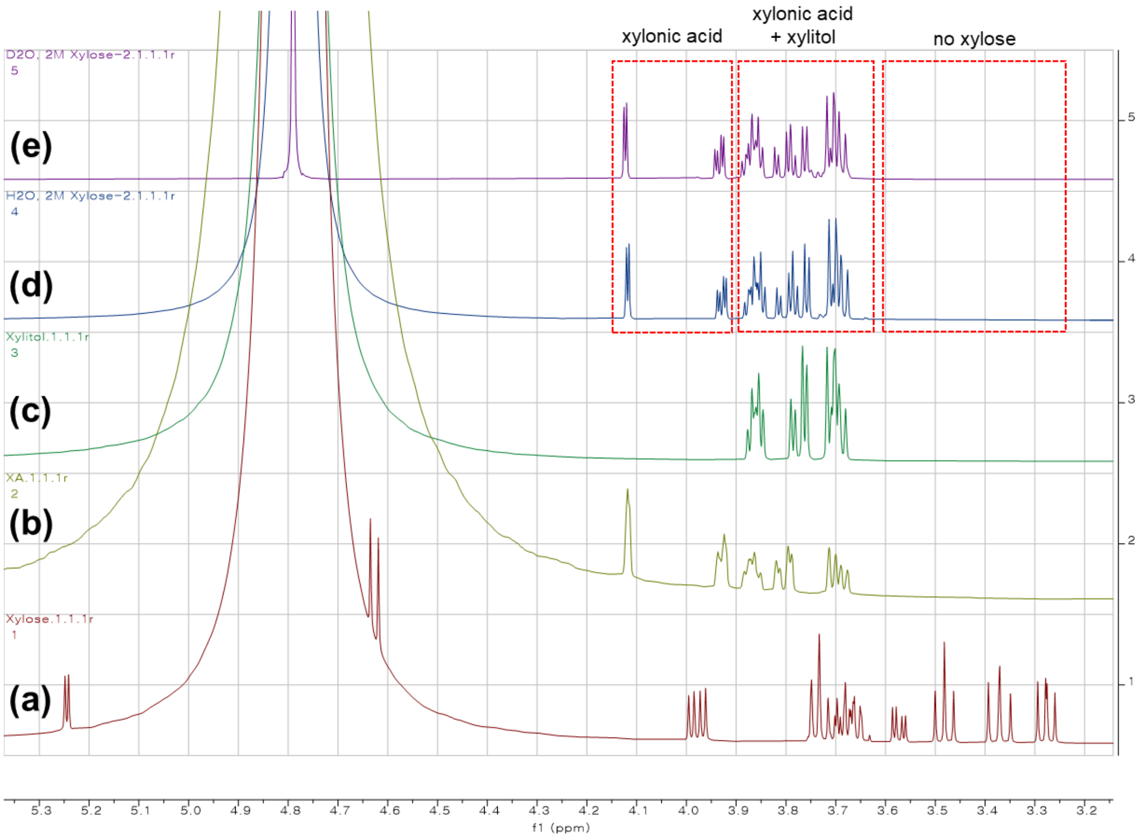

Figure S7. NMR spectra of (a) xylose, (b) xylonic acid, (c) xylitol, and (d) reaction product with H_2O as a solvent, and (e) reaction product with D_2O as a solvent (2 M xylose, 2 M KOH, 0.4 g Pt/ZrO_2, 25 °C, 6 mL H_2O).

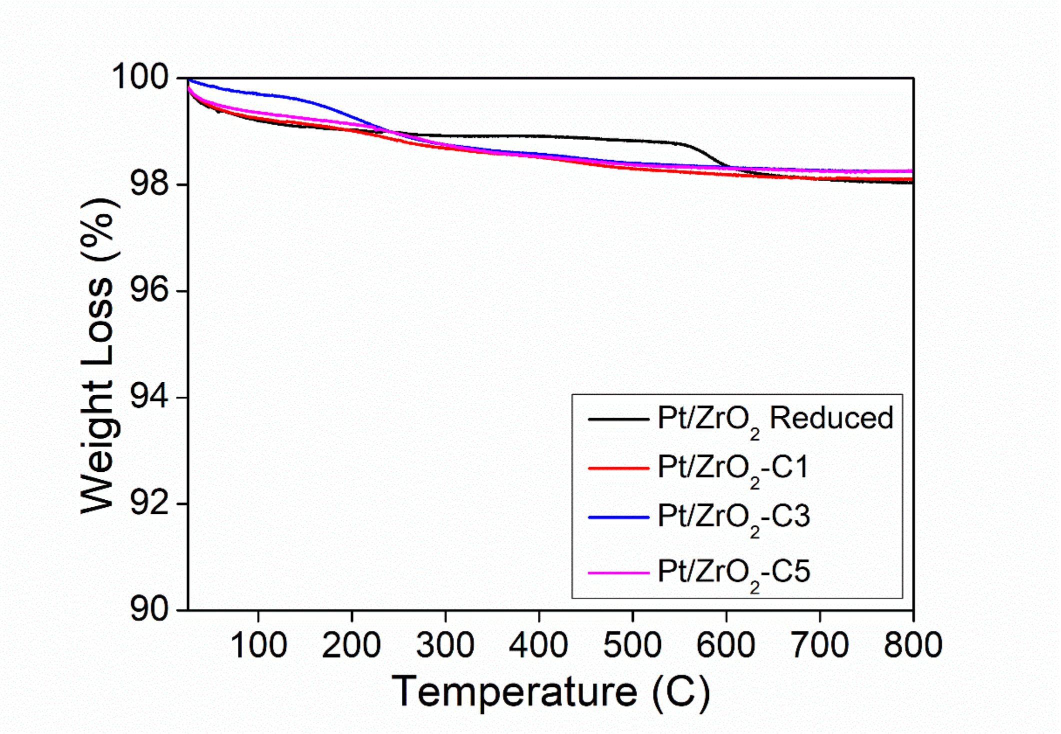

Figure S8. TGA results of 5%Pt/ZrO_2 before reaction and after reactions (cycle 1, cycle 3, and cycle 5).

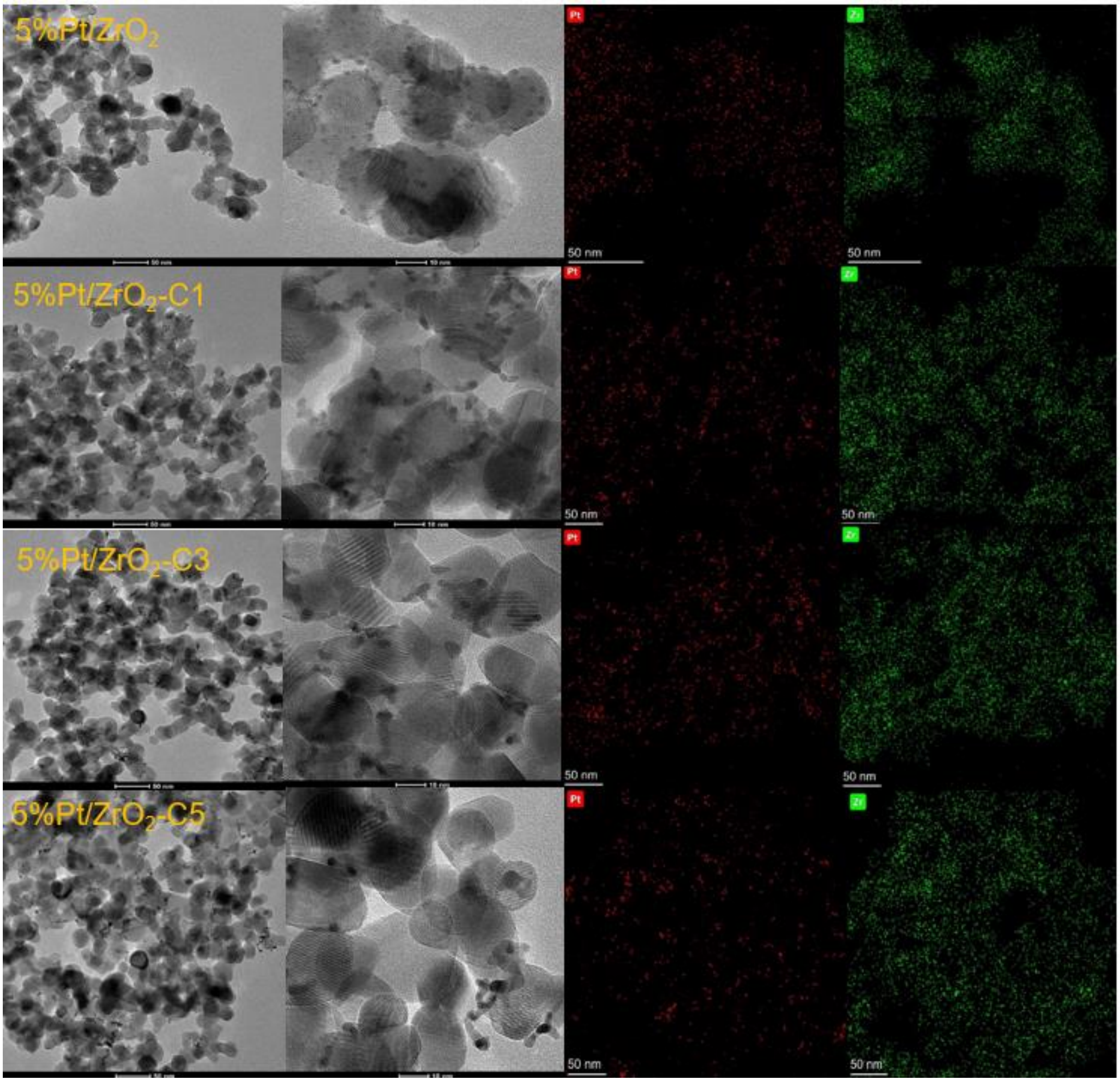

Figure S9. TEM images and EDS maps of 5%Pt/ZrO_2 before reaction and after reactions (cycle 1, cycle 3, and cycle 5). Scale bar = 50 nm and 10 nm.

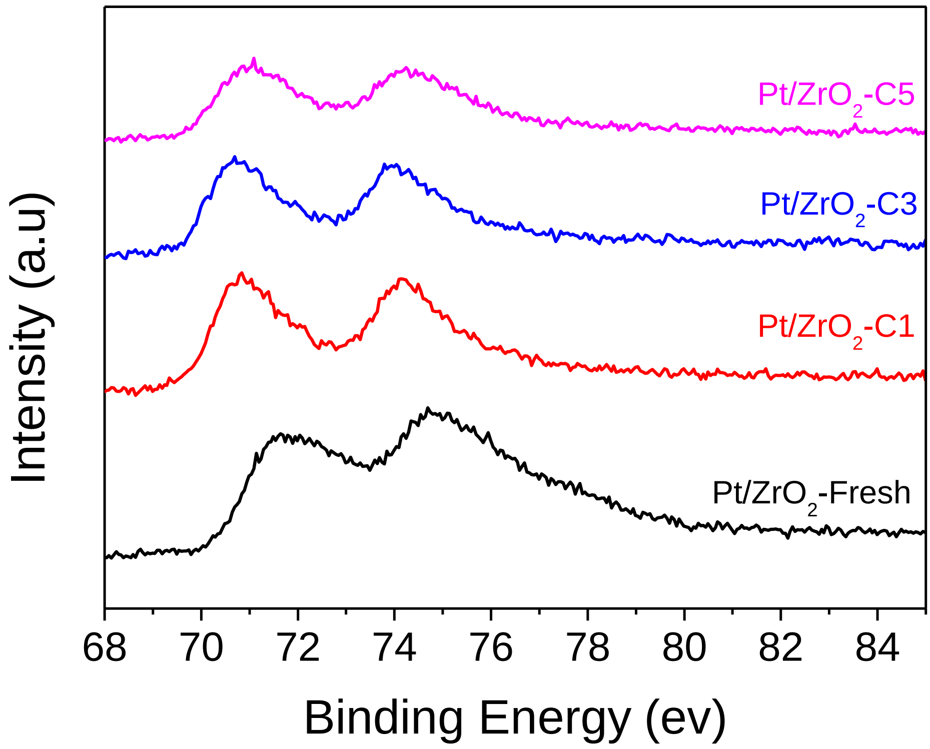

Figure S10. Pt 4f XPS spectra of 5% Pt/ZrO_2 before reaction and after reactions (cycle 1, cycle 3, and cycle 5).

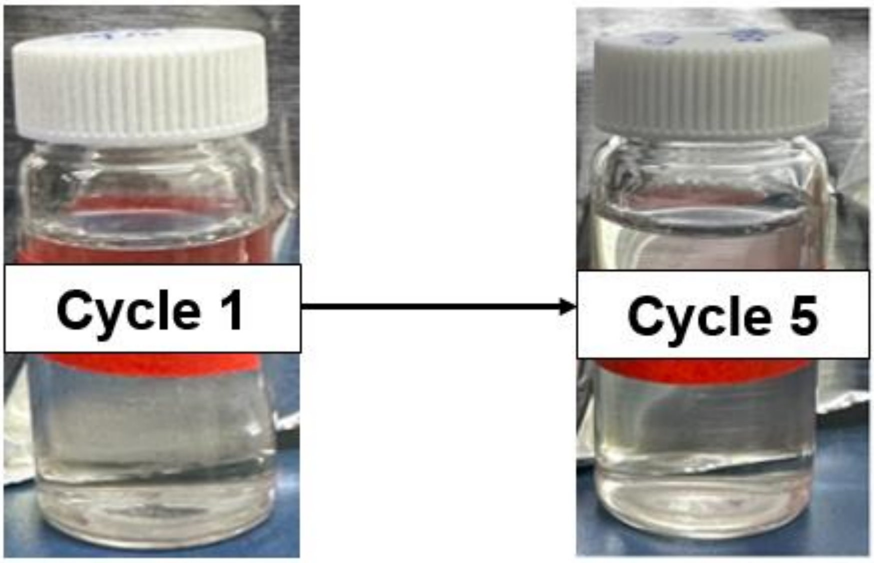

Figure S11. Photograph of reaction product solutions after cycle 1 and cycle 5 using 5% Pt/ZrO_2 catalyst.

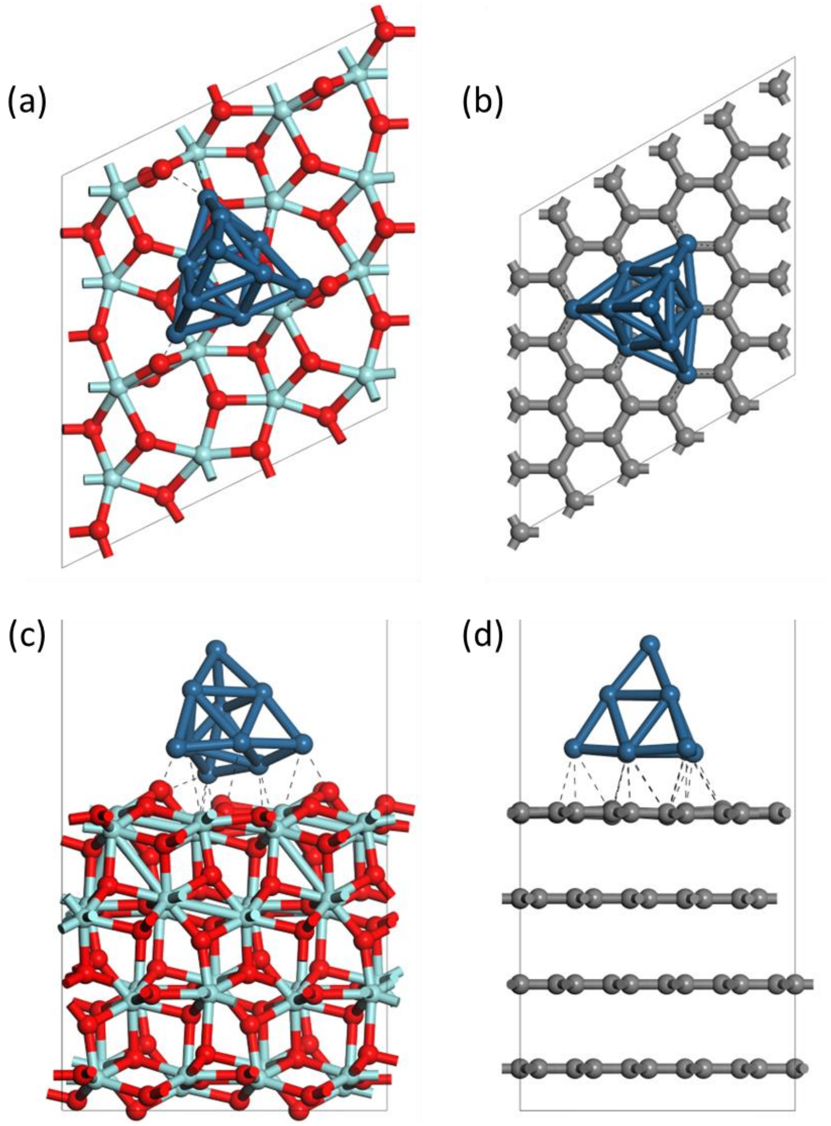

Figure S12. Top and side views of optimized adsorption structures of Pt_{10} (a and c) on monoclinic $ZrO_2(111)$ and (b and d) on graphite. For the top view images, only the first support layer is depicted for clarity. The interfacial close contacts, which are estimated using the scaled sum of van der Waals radii, are indicated with dashed lines.

a* → **b**, ΔE = –1.56 eV

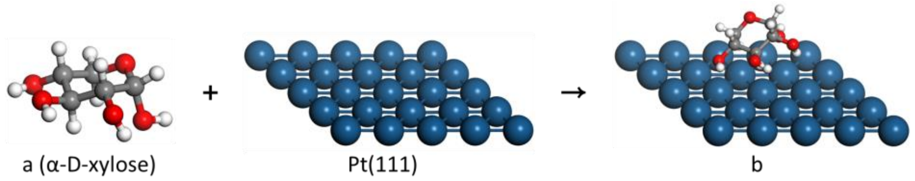

b → **c** (+ H)**, ΔE = +0.67 eV

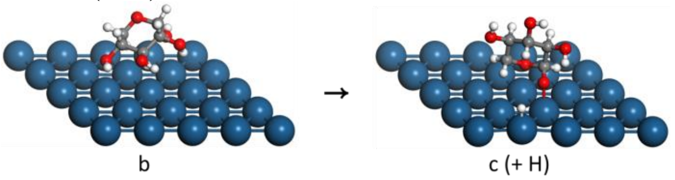

c → **d** (+ H), ΔE = –0.47 eV

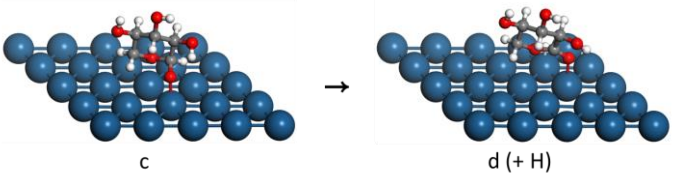

d (+ OH) → **e**, ΔE = –0.10 eV

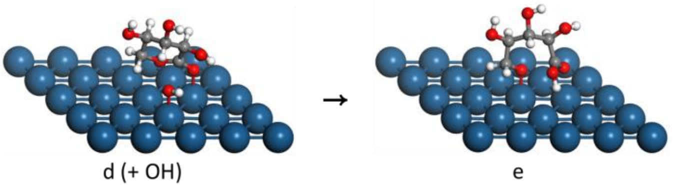

e (+ H) → **f**, ΔE = –0.13 eV

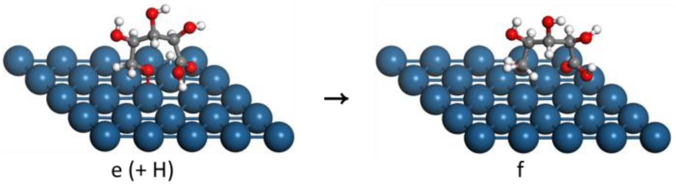

f → **g**, ΔE = +1.15 eV

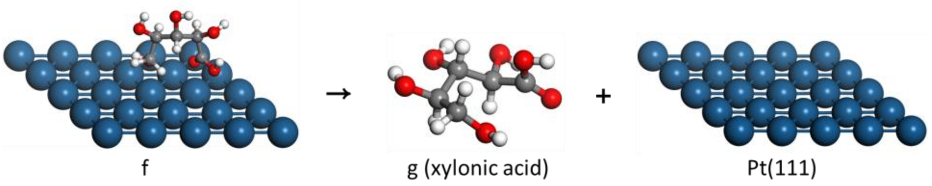

Figure S13. The energy changes for each reaction step from xylose to xylonic acid (see **Figure 8** in main text). For D-xylose, α form is more stable than β by 0.07 eV as reported in previous study[S5]. H or OH species in parentheses, which is attached or removed from the molecule during reaction step, is additionally adsorbed on Pt(111) surface.

a* → **b**, ΔE = –1.56 eV

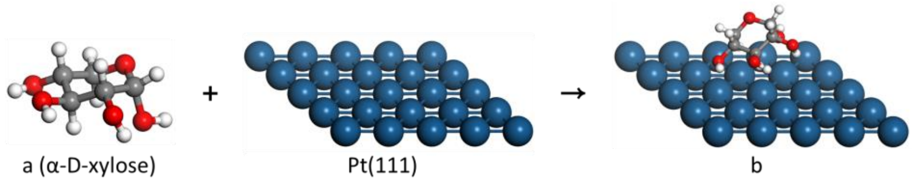

a (α-D-xylose) + Pt(111) → b

b → **c** (+ H)**, ΔE = +0.67 eV

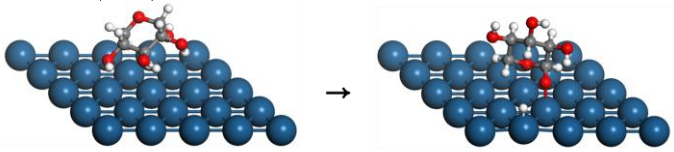

b → c (+ H)

c (+ H) → **h**, ΔE = +0.22 eV

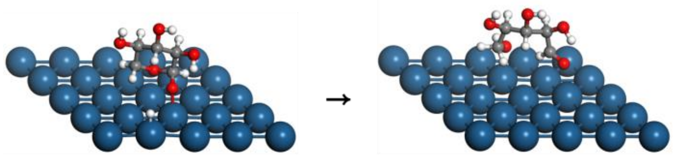

c (+ H) → h

h (+ H) → **i**, ΔE = –0.57 eV

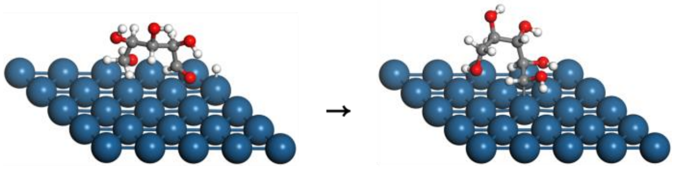

h (+ H) → i

i (+ H) → **j**, ΔE = +0.45 eV

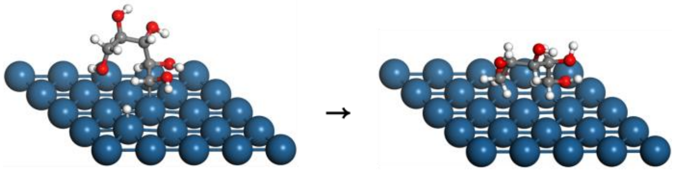

i (+ H) → j

j → **k**, ΔE = +1.24 eV

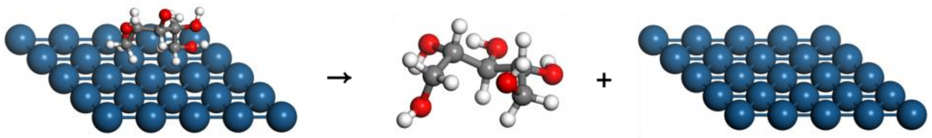

j → k (xylitol) + Pt(111)

Figure S14. The energy changes for each reaction step from xylose to xylitol (see **Figure 8** in main text). For D-xylose, α form is more stable than β by 0.07 eV as reported in previous study[S5]. H or OH species in parentheses, which is attached or removed from the molecule during reaction step, is additionally adsorbed on Pt(111) surface.

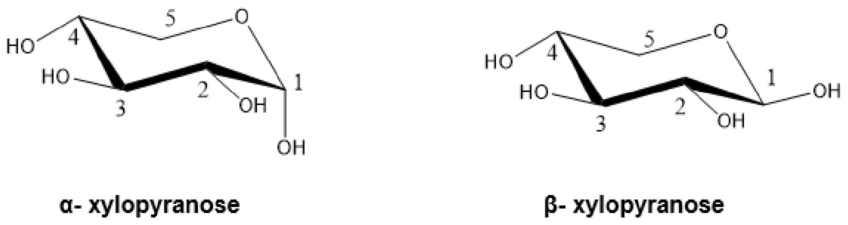

Figure S15. Xylose structures with carbon numbers for better understanding on adsorption on Pt surface.

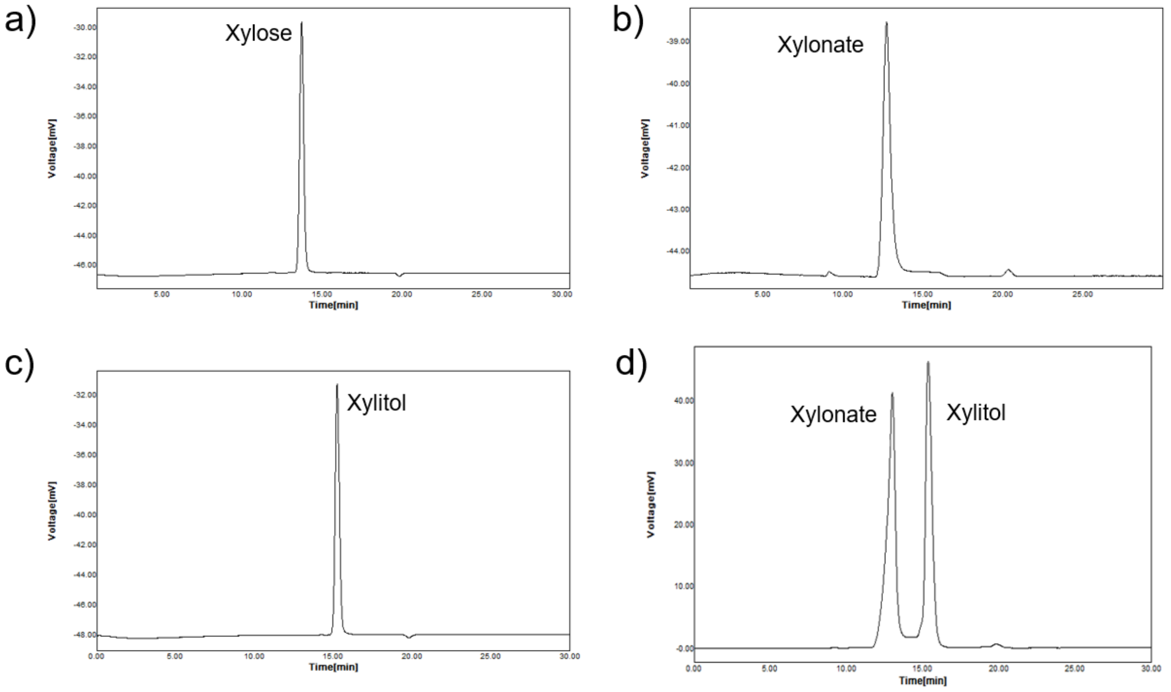

Figure S16. HPLC curves for a) xylose, b) lithium xylonate, c) xylitol standards, and d) reaction product at 100% conversion. A small peak at 20 min is from the mobile phase.

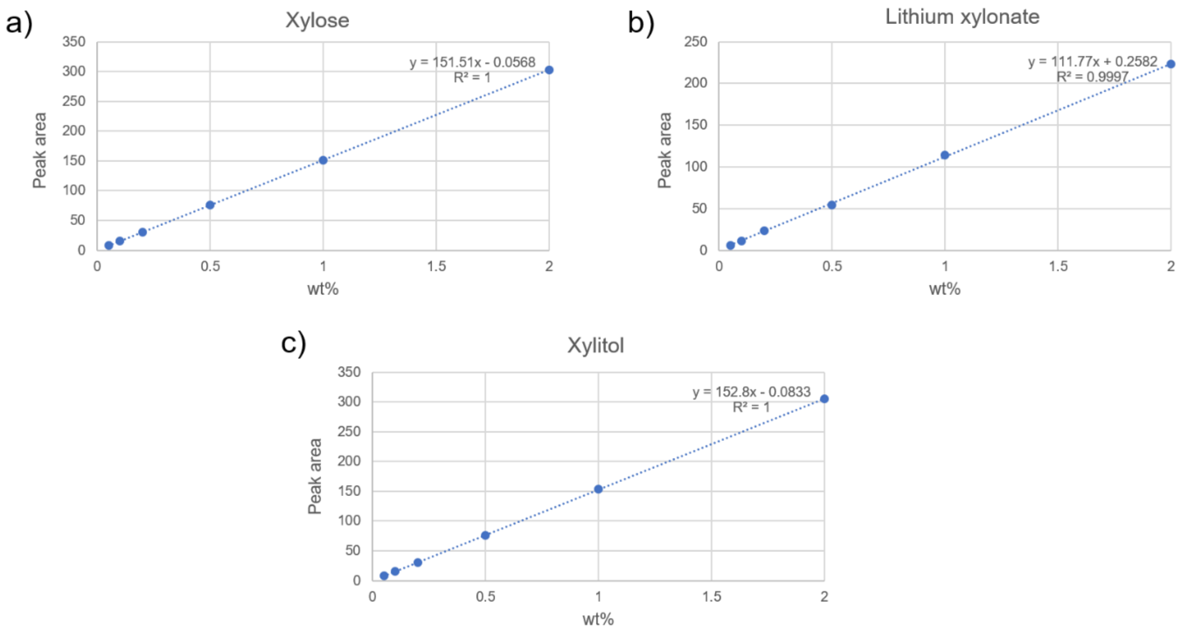

Figure S17. Calibration curves for a) xylose, b) lithium xylonate, and c) xylitol.

Table S1. Xylose conversion over various reaction conditions.

Entry	Conditions	Catalyst	Conv. (%)	Xylonic acid/Xylitol yield (%)	Product sample
1	KOH 2 M	-	>5	0/0	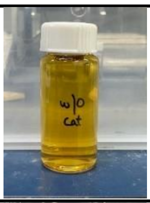
2	No base (pH 7)	Pt/ZrO_2	0	0/0	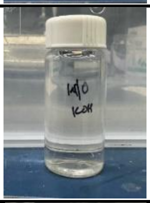
3	H_2SO_4 (pH 1)	Pt/ZrO_2	>50	0/0	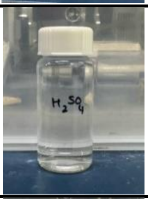
4	10 bar N_2, KOH 2 M	Pt/ZrO_2	100	50/50	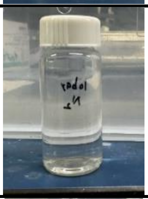
5	H_2O	Pt/ZrO_2	100	50/50	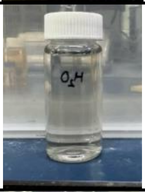
6	D_2O	Pt/ZrO_2	100	50/50	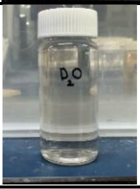
